# Epstein–Barr Virus‐Encoded *MicroRNA‐BART18‐3p* Promotes Colorectal Cancer Progression by Targeting De Novo Lipogenesis

**DOI:** 10.1002/advs.202202116

**Published:** 2022-10-28

**Authors:** Qingtao Meng, Hao Sun, Shenshen Wu, Giuseppe Familiari, Michela Relucenti, Michael Aschner, Xiaobo Li, Rui Chen

**Affiliations:** ^1^ Beijing Key Laboratory of Environmental Toxicology, School of Public Health Capital Medical University Beijing 100069 P. R. China; ^2^ Department of Oncology Capital Medical University Beijing 100069 P. R. China; ^3^ Department of Occupational Health School of Public Health Shanxi Medical University Taiyuan 030001 China; ^4^ Key Laboratory of Environmental Medicine Engineering Ministry of Education School of Public Health Southeast University Nanjing 210009 P. R. China; ^5^ Laboratory of Electron Microscopy “Pietro Motta” SAIMLAL Department Faculty of Pharmacy and Medicine Sapienza University of Rome via Alfonso Borelli 50 Rome 00161 Italy; ^6^ Department of Molecular Pharmacology Albert Einstein College of Medicine Forchheimer 209, 1300 Morris Park Avenue Bronx NY 10461 USA; ^7^ Advanced Innovation Center for Human Brain Protection Capital Medical University Beijing 100069 P. R. China; ^8^ Institute for Chemical Carcinogenesis Guangzhou Medical University Guangzhou 511436 P. R. China

**Keywords:** colorectal cancer (CRC), de novo lipogenesis, Epstein–Barr virus (EBV), lactate dehydrogenase A (LDHA), microRNA

## Abstract

The Epstein–Barr virus (EBV) genome encodes a cluster of 22 viral microRNAs, called miR‐BamHI‐A rightward transcripts (miR‐BARTs), which are shown to promote the development of cancer. Here, this study reports that *EBV‐miR‐BART18‐3p* is highly expressed in colorectal cancer (CRC) and is closely associated with the pathological and advanced clinical stages of CRC. Ectopic expression of *EBV‐miR‐BART18‐3p* leads to increased migration and invasion capacities of CRC cells in vitro and causes tumor metastasis in vivo. Mechanistically, *EBV‐miR‐BART18‐3p* activates the hypoxia inducible factor 1 subunit alpha/lactate dehydrogenase A axis by targeting Sirtuin, which promotes lactate accumulation and acetyl‐CoA production in CRC cells under hypoxic condition. Increased acetyl‐CoA utilization subsequently leads to histone acetylation of fatty acid synthase and fatty acid synthase‐dependent fat synthesis, which in turn drives de novo lipogenesis. The oncogenic role of *EBV‐miR‐BART18‐3p* is confirmed in the patient‐derived tumor xenograft mouse model. Altogether, the findings define a novel mechanism of *EBV‐miR‐BART18‐3p* in CRC development through the lipogenesis pathway and provide a potential clinical intervention target for CRC.

## Graphical abstract

*EBV‐miR‐BART18‐3p* is highly expressed in colorectal cancer (CRC) and is closely associated with the pathological and advanced clinical stages of CRC. *EBV‐miR‐BART18‐3p* promotes histone acetylation and enhances de novo lipogenesis by upregulating lactate dehydrogenase A‐mediated metabolic processes and the fatty acid synthase‐mediated de novo lipogenesis pathway. Maintaining the homeostasis of lipogenesis effectively prevents CRC aggravation and metastasis caused by Epstein–Barr virus infection.

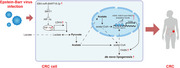

## 1 Introduction

Colorectal cancer (CRC) is a major cause of cancer‐related mortality globally, resulting in more than 1 million incidences and 550 000 deaths in 2018.^[^ ^1^ ^]^ The occurrence and development of CRC are affected by diverse factors, including heredity, environmental exposure, lifestyle, and intestinal inflammation. Infection and interference of the exogenous genome have also been correlated with CRC incidence.^[^ ^2^, ^3^, ^4^ ^]^ Epstein–Barr virus (EBV), a well‐recognized oncogenic virus, persistently infects over 90% of the world's population, often in an asymptomatic manner. It was the first virus identified as a group I carcinogen by the International Agency for Research on Cancer.^[^ ^5^, ^6^ ^]^ In addition to causing epithelial herpes, it is closely involved in the development of cancers, such as Burkitt's lymphoma, nasopharyngeal carcinoma, and gastric carcinoma, all of which are classified as EBV‐related tumors.^[^ ^7^, ^8^ ^]^ At the latent stage, EBV in cancer cells promotes immune evasion while remaining transcriptionally active. Switching of EBV between latent and lytic stages is dependent upon the differential expression of surface proteins in infected cell membranes.^[^ ^9^ ^]^ BamHI‐A rightward transcripts (BARTs) and EBV‐encoded RNAs are the most widely expressed viral transcripts in the latent stage.^[^ ^10^ ^]^ Intensive research efforts have focused on EBV‐encoded RNAs, such as EBNA, which encodes and expresses latent membrane proteins including LMP1, LMP2A, and LMP2B.^[^ ^11^ ^]^ In addition to virus‐encoded proteins, EBV‐encoded miRNAs (EBV‐miRs) play an indispensable role in the pathogenesis and progression of EBV‐associated tumors.^[^ ^11^, ^12^ ^]^

miRNAs are a series of noncoding single‐strand RNAs comprising 17–23 nucleotides. A total of 25 EBV‐miR precursors that produce 48 mature miRNAs have been recognized. Most mature miRNAs are located in the BART region of the EBV genome, which contains 22 miRNA precursors, including *EBV‐miR‐BART1‐22*.^[^ ^13^, ^14^ ^]^ Though several studies have reported the oncogenic role of *EBV‐miR‐BARTs* in gastric carcinoma and nasopharyngeal carcinoma,^[^ ^15^, ^16^, ^17^, ^18^, ^19^ ^]^ few studies have investigated their roles in CRC.

Metabolically, cancer cells acquire energy and nutrients from hypoxic and nutrient‐poor microenvironments and subsequently utilize them for biosynthesis, cell viability maintenance, growth and uncontrolled proliferation.^[^ ^20^ ^]^ Enhanced metabolism produces several intermediate metabolites, such as acetyl‐CoA and fatty acids, which are required for the survival, rapid proliferation, and increased malignant phenotypes of cancer cells,^[^ ^21^ ^]^ which leads to tumor progression.^[^ ^22^ ^]^ However, the association between *EBV‐miR‐BARTs* and metabolic reprogramming in CRC cells remains obscure.

In this study, we discovered that hyperexpression of the EBV‐encoded miRNA, *EBV‐miR‐BART18‐3p*, promoted progression of CRC through activation of a de novo lipogenesis pathway both in vitro and in vivo. Our findings provide new insights into the role of exogenous microRNAs in CRC development and support a novel strategy for clinical intervention.

## 2 Results

### 2.1 *EBV‐miR‐BART18‐3p* Expression Is Associated with CRC Progression

**Figure 1:**
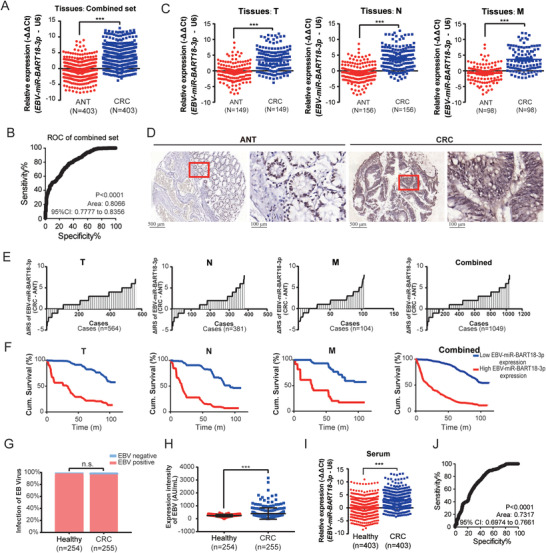
*EBV‐miR‐BART18‐3p* expression is significantly associated with CRC progression. A) *EBV‐miR‐BART18‐3p* expression levels in fresh tissues from the combined set of patients for CRC (CRC) and adjacent noncancerous (ANT). Data are presented as −ΔΔCt (*t*‐test, three technical replicates each). B) Receiver operating (ROC) curve of *EBV‐miR‐BART18‐3p* to evaluate its ability to distinguish CRC and ANT tissues. C) The expression levels of *EBV‐miR‐BART18‐3p* in CRC or ANT tissues with T, N, or M stages (T: primary tumor; N: tumor with regional lymph node involvement; M: distant metastasis). Data are presented as −ΔΔCt (*t*‐test, three technical replicates each). D) Representative images of *EBV‐miR‐BART18‐3p* expression in tissue microarrays (TMAs). E) Immune reactive scores (IRS) for *EBV‐miR‐BART18‐3p* staining in TMAs. F) Kaplan–Meier curves showing the overall survival of CRC patients according to the tumoral *EBV‐miR‐BART18‐3p* expression levels in TMAs (log‐rank test). G) EBV prevalence rates of CRC patients and matched healthy donors (*t*‐test). H) EBV titers of CRC patients and matched healthy donors (*t*‐test). I) *EBV‐miR‐BART18‐3p* levels in the serum of CRC patients and matched healthy donors (*t*‐test, three technical replicates each). J) ROC curves to evaluate the diagnostic value of serum *EBV‐miR‐BART18‐3p* in CRC. **P < 0.01, ***P < 0.001.

In our previous study,^[^ ^23^ ^]^ we performed miRNA microarray analysis of six pairs of fresh colorectal cancer (CRC) and adjacent noncancerous tissues (ANT), as well as six colorectal adenoma (ADE) tissues (Table S1, Supporting Information). Three differentially expressed miRNAs were identified among the three types of specimens: *EBV‐miR‐BART18‐3p*, *has‐miR‐135a‐5p*, and *has*‐*miR‐204‐5p* (Table S2, Supporting Information). *EBV‐miR‐BART18‐3p* was the most prominently upregulated gene in CRC, with progressively higher expression in CRC than in ADE or ANT (Figure S1A–C, Supporting Information). To verify the upregulation of *EBV‐miR‐BART18‐3p* expression in CRC, we evaluated its expression levels in fresh CRC tissues from testing and validation cohorts (*n* = 322 and 81, respectively) (Table S3, Supporting Information). The *EBV‐miR‐BART18‐3p* expression levels were dramatically increased in CRC tissues compared to ANT in the testing, validation, and combined sets (*P* < 0.0001 in each set) (**Figure** **1**A and Figure S2A, Supporting Information). Furthermore, receiver operating characteristic (ROC) analysis demonstrated that *EBV‐miR‐BART18‐3p* expression could accurately distinguish CRC from ANT (area under the ROC curve = 0.8036 [*P* < 0.0001] in the testing set, 0.8208 [*P* < 0.0001] in the validation set, and 0.8066 [*P* < 0.0001] in the combined set) (Figure 1B and Figure S2B, Supporting Information).

To elucidate the relationship between *EBV‐miR‐BART18‐3p* expression and pathological features of CRC, we performed stratified analysis of the CRC cohorts according to tumor node metastasis (TNM) stage information. As shown in Figure 1C, *EBV‐miR‐BART18‐3p* expression levels were markedly elevated in CRC tissues compared to ANT, at T, N, and M stages. As additional confirmation, we evaluated tissue microarrays (TMAs), which included 1078 CRC tissues and corresponding ANT with ten‐year follow‐up survival information (Table S4, Supporting Information). A significantly higher level of *EBV‐miR‐BART18‐3p* was observed in CRC tissues compared with the corresponding ANT (Figure 1D,E and Figure S2C, Supporting Information). Patients with higher *EBV‐miR‐BART18‐3p* levels in tumors also displayed unfavorable pathological parameters, including increased advanced cancer rates (Table S5, Supporting Information) and shorter overall survival times (Figure 1F, *P* < 0.001, and Figure S2D, Supporting Information). Furthermore, multivariate Cox regression analysis indicated that *EBV‐miR‐BART18‐3p* expression serves as an indicator of CRC prognosis (Table S6, Supporting Information).

Because *EBV‐miR‐BART18‐3p* is encoded by EBV, we sought to investigate whether the prevalence of EBV infection varies between CRC patients and healthy donors. The results demonstrated that the EBV infection rates were indistinguishable in 255 CRC patients versus 254 healthy donors; however, titers of EBV were dramatically elevated in CRC patient serum (average EBV titers = 399.7) compared to serum from healthy controls (average EBV titers = 243.9, *P* < 0.0001) (Figure 1G,H). Furthermore, serum *EBV‐miR‐BART18‐3p* expression levels were significantly increased in CRC patients compared with healthy donors and could accurately discriminate CRC patients from healthy donors as evaluated by ROC analysis (Figure 1I). Stratified analysis also indicated that serum *EBV‐miR‐BART18‐3p* levels were elevated in CRC patients compared to matched healthy donors at each TNM stage (Figure S3, Supporting Information). Taken together, our results establish the association between elevated *EBV‐miR‐BART18‐3p* and CRC progression and suggest that both tumor and serum *EBV‐miR‐BART18‐3p* expression levels could serve as prognostic biomarkers.

### 2.2 *EBV‐miR‐BART18‐3p* Enhances CRC Malignant Phenotypes In Vitro and In Vivo

**Figure 2:**
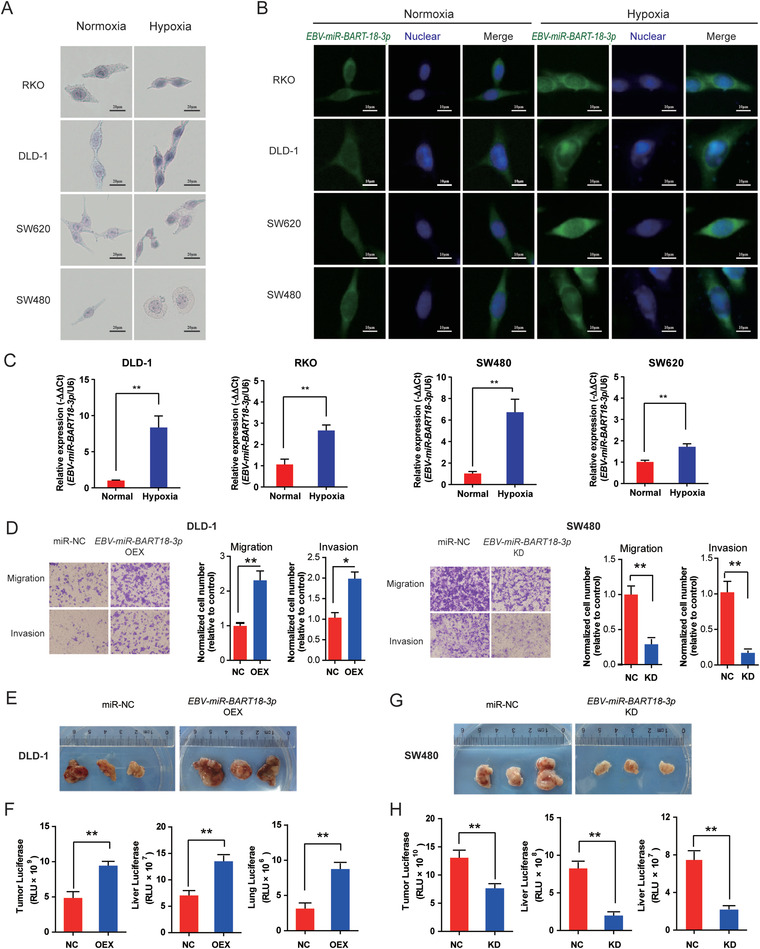
*EBV‐miR‐BART18‐3p* enhances the malignant phenotype of CRC in vitro and in vivo. A) In situ hybridization and B) fluorescent in situ hybridization assays showing the expression levels and intracellular localization of *EBV‐miR‐BART18‐3p* in CRC cells. C) *EBV‐miR‐BART18‐3p* expression levels in CRC cells under normoxic or hypoxic conditions as evaluated by PCR. Data are presented as −ΔΔCt (*t*‐test, *n* = 3/group). D) Migratory and invasive capacities in *EBV‐miR‐BART18‐3p* NC versus KD or NC versus OEX CRC cells. (*t*‐test, *n* = 3/group). E) Representative images of xenografts from nude mice with subcutaneous *EBV‐miR‐BART18‐3p* NC or OEX DLD‐1 cell injections. F) Luciferase activity in tumor, liver, and lung tissues from nude mice with DLD‐1 injections (*t*‐test, *n* = 3/group). G) Representative images of xenografts from nude mice with subcutaneous *EBV‐miR‐BART18‐3p* NC or KD SW480 cell injection. H) Luciferase activity in tumor, liver, and lung tissues from nude mice with SW480 injections (*t*‐test, *n* = 3/group). *P < 0.05, **P < 0.01.

To further evaluate *EBV‐miR‐BART18‐3p* function, we examined its expression levels in multiple CRC cell lines and selected SW480 and RKO (with relatively high expression levels), and DLD‐1 and SW620 (with relatively low expression levels), for further study (Figure S4, Supporting Information). We then retained these cells under normoxia conditions or subjected them to hypoxia, which is known to facilitate the development of CRC^[^ ^24^, ^25^, ^26^, ^27^, ^28^, ^29^, ^30^ ^]^). In situ hybridization (**Figure** **2**A) and fluorescent in situ hybridization (Figure 2B) assays demonstrated that *EBV‐miR‐BART18‐3p* was predominantly localized to the cytoplasm, with some expression in the nucleus, and that under hypoxic conditions, *EBV‐miR‐BART18‐3p* expression levels were dramatically increased. The increased levels of *EBV‐miR‐BART18‐3p* in each of the four CRC cell lines in response to hypoxia were further verified by PCR (Figure 2C).

Next, we knocked down *EBV‐miR‐BART18‐3p* (KD) in SW480 and RKO and overexpressed *EBV‐miR‐BART18‐3p* (OEX) in DLD‐1 and SW620 cells using lentivirus‐mediated transfection. *EBV‐miR‐BART18‐3p* OEX dramatically increased the cellular migration and invasion capacities in DLD‐1 and SW620 cells, while *EBV‐miR‐BART18‐3p* KD impaired the malignant phenotypes of SW480 and RKO cells (Figure 2D and Figure S5A,B, Supporting Information).

To determine whether the malignant function of *EBV‐miR‐BART18‐3p* can also be observed in vivo, we injected CRC cells subcutaneously in nude mice. *EBV‐miR‐BART18‐3p* OEX significantly increased the tumor size and metastasis of CRC to the liver and lungs (Figure 2E,F); while *EBV‐miR‐BART18‐3p* KD alleviated malignant progression (Figure 2G,H, and Figure S5C–H, Supporting Information). Taken together, these results indicate that *EBV‐miR‐BART18‐3p* enhances proliferation and metastasis of CRC cells both in vitro and in vivo.

### 2.3 *EBV‐miR‐BART18‐3p* and Lactate Dehydrogenase A are Coexpressed within a CRC Network

**Figure 3:**
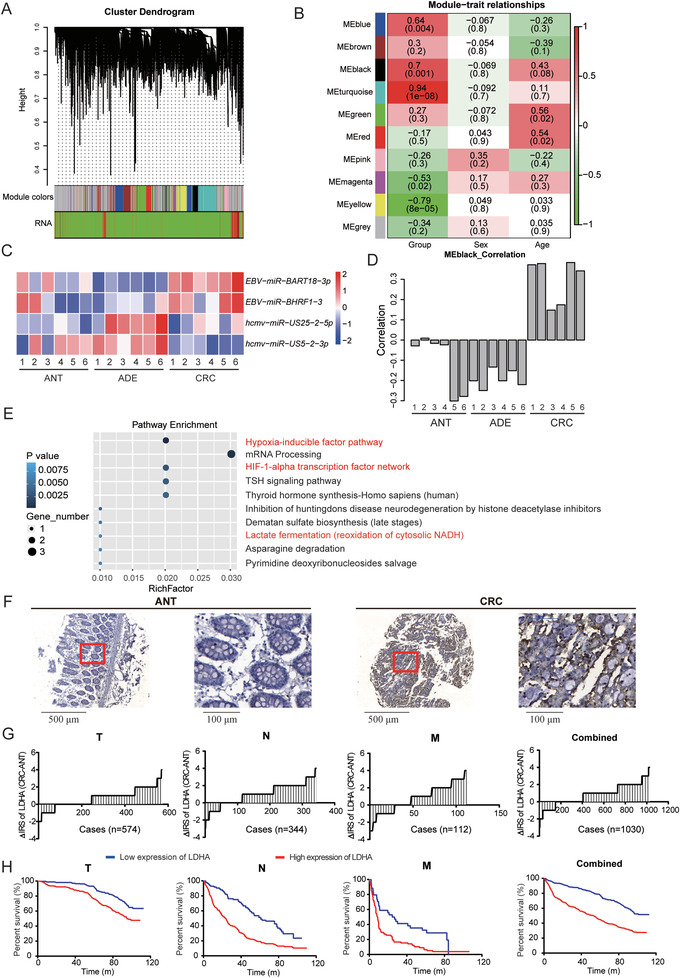
Coexpression of *EBV‐miR‐BART18‐3p* and LDHA in CRC as identified by WGCNA analysis. A) Cluster dendrogram of modules identified by WGCNA based on mRNA and miRNA microarray data. B) The associations between each module and clinical characteristics. Red indicates positive correlations and green indicates negative correlations. Numbers in each lattice indicate the correlation coefficient (the *P*‐value of the correlation). C) Heatmap showing the abundance of significantly differentially expressed exogenous miRNAs in the WGCNA model. D) Histogram showing the overall gene expression patterns in the black module of ANT, ADE, and CRC. E) Top 10 KEGG enrichment pathways of mRNAs in the black module. F) Representative images of LDHA expression in TMAs. G) Change in immune reactivity score (ΔIRS) of LDHA expression in TMAs. H) Kaplan–Meier curves showing the overall survival of CRC patients according to tumoral LDHA expression levels in TMAs (log‐rank test).

To evaluate the underlying mechanism of *EBV‐miR‐BART18‐3p* in CRC, we performed weighted gene coexpression network analysis (WGCNA) as a bioinformatics tool to identify key molecules responsible for specific clinical traits.^[^ ^31^, ^32^ ^]^ We constructed networks of differentially expressed mRNA^[^ ^33^ ^]^ and miRNA from CRC microarray data (**Figure** **3**A). A total of ten coexpression modules were identified, and five of these modules (blue, black, turquoise, magenta, and yellow) were significantly correlated with CRC progression but showed no association with gender and age (Figure 3B). Furthermore, *EBV‐miR‐BART18‐3p* was one of only four exogenous microRNAs that were identified within these five WGCNA modules. Among the four exogenous microRNAs, *EBV‐miR‐BART18‐3p* and *EBV‐miR‐BHRF1‐3* were located in the MEblack module (correlation coefficient = 0.7, *P* = 0.001); and *hcmv‐miR‐US25‐2‐5p* and *hcmv‐miR‐US5‐2‐3p* were located in the MEred module (correlation coefficient = −0.17, *P* value = 0.5). The relative expression levels of these microRNAs in ANT, ADE, and CRC tissues are shown in a heatmap (Figure 3C).

To verify the key role of *EBV‐miR‐BART18‐3p* within the mRNA‐miRNA network, we repeated WGCNA analysis once more after excluding *EBV‐miR‐BART18‐3p* from the MEblack module. In the new WGCNA network, 52.5% of the genes previously belonging to the MEblack module were transferred to the MEturquoise module, which showed no statistical correlation with CRC development (correlation coefficient = 0.16, *P* value = 0.5) (Figure S6, Supporting Information). These results corroborate the significance of the *EBV‐miR‐BART18‐3p*–mRNA network in the original MEblack module (Figure 3D).

To further examine the function of MEblack, we performed Kyoto Encyclopedia of Genes and Genomes (KEGG) pathway enrichment analysis. Notably, several cancer‐related pathways that contain the lactate dehydrogenase A (LDHA) gene were enriched, including the hypoxia‐inducible factor (HIF) pathway, the HIF‐1*α* transcription factor network, and the lactate fermentation pathway (Figure 3E and Table S7, Supporting Information). These findings are consistent with a role for LDHA as a key enzyme in tumor metabolism.^[^ ^34^, ^35^ ^]^ Moreover, in TMAs, a dramatic elevation of LDHA expression was observed in CRC compared to corresponding ANT tissues (Figure 3F,G). Patients with high LDHA levels in tumors also displayed unfavorable pathological parameters, such as advanced cancer grade (Table S8, Supporting Information) and shorter overall survival times (Figure 3H and Table S9, Supporting Information). Taken together, these results suggest that *EBV‐miR‐BART18‐3p* and LDHA are critically involved in CRC development via the *EBV‐miR‐BART18‐3p*–mRNA network.

### 2.4 *EBV‐miR‐BART18‐3p* Regulates Metabolic Pathways and Acetyl‐CoA Production by Upregulating LDHA

**Figure 4:**
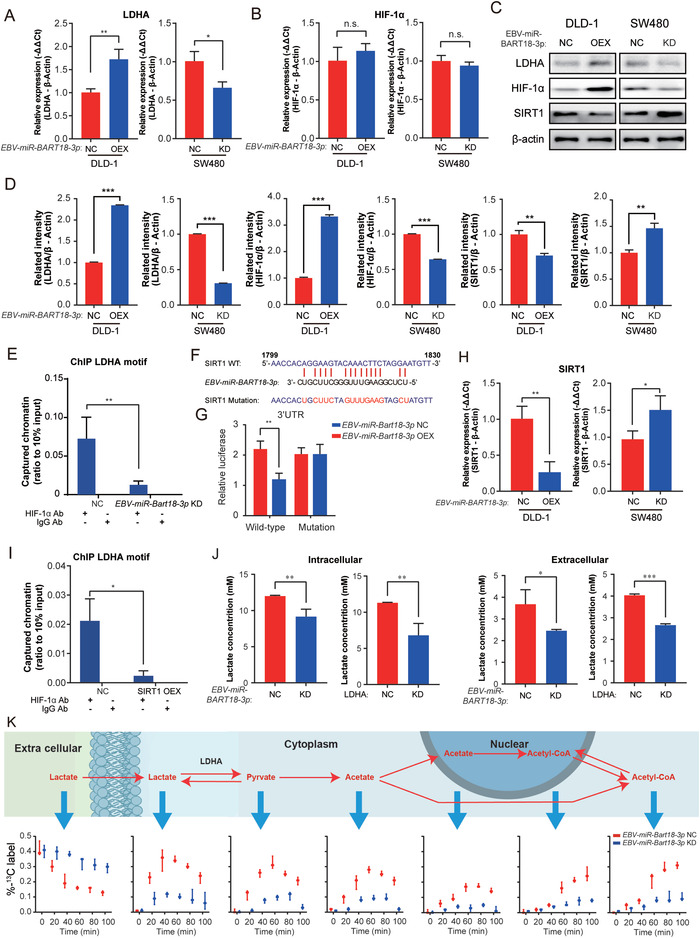
*EBV‐miR‐BART18‐3p* regulates metabolic pathways and acetyl‐CoA production by upregulating LDHA. A) LDHA and B) HIF‐1*α* mRNA expression levels in hypoxia‐treated CRC cells (*EBV‐miR‐BART18‐3p* NC vs KO; or *EBV‐miR‐BART18‐3p* NC vs OEX) as evaluated by PCR. Data are presented as −ΔΔCt (*t*‐test, *n* = 3/group). C) Western blot of LDHA, HIF‐1*α*, and SIRT1 protein levels in hypoxia‐treated CRC cells (*EBV‐miR‐BART18‐3p* NC vs KD; or *EBV‐miR‐BART18‐3p* NC vs OEX). D) Quantification of western blot results (*t*‐test, three technical replicates each). E) LDHA ChIP assays using HIF‐1*α* antibody in hypoxia‐treated SW480 cells (two‐way ANOVA, three technical replicates each). F) Predicted site for *EBV‐miR‐BART18‐3p* binding in the SIRT1 3’UTR; wild‐type (WT) and mutant sequences are indicated. G) Relative luciferase activities of hypoxia‐treated DLD‐1 cells expressing SIRT1 WT or mutant luciferase reporter plasmids and transfected with *EBV‐miR‐BART18‐3p* or NC mimics (*t*‐test, *n* = 3/group). (H) SIRT1 mRNA expression levels in hypoxia‐treated CRC cells (*EBV‐miR‐BART18‐3p* NC versus KD; or *EBV‐miR‐BART18‐3p* NC vs OEX) as evaluated by PCR. Data are presented as −ΔΔCt (*t*‐test, *n* = 3/group). I) LDHA ChIP assays using HIF‐1*α* antibody in hypoxia‐treated SW480 cells (two‐way ANOVA, three technical replicates each). J) The concentration of lactate in hypoxia‐treated SW480 cells and culture medium (*t*‐test, three technical replicates each). K) Schematic depicting the flux of ^13^C‐labeled lactate to acetyl‐CoA. The histograms show the flux of ^13^C in hypoxia‐treated *EBV‐miR‐BART18‐3p* NC and KD SW480 cells. n.s.: no significance, *P < 0.05, **P < 0.01, ***P < 0.001.

HIF‐1*α* facilitates tumor cell adaptation to the hypoxic microenvironment^[^ ^36^ ^]^ and has been reported to activate LDHA expression by binding a hypoxia response element (HRE) within its promoter.^[^ ^37^ ^]^ Thus, to determine whether a *EBV‐miR‐BART18‐3p*‐dependent increase in LDHA expression in CRC is mediated by HIF‐1*α*, we evaluated the mRNA expression levels of LDHA and HIF‐1*α* after EBV‐*miR‐BART18‐3p* overexpression and knockdown. *EBV‐miR‐BART18‐3p* OEX significantly increased LDHA but not HIF‐1*α* mRNA levels in hypoxic CRC cells (LDHA: *P* = 0.0056, HIF‐1*α*: *P* = 0.2907) (**Figure** **4**A). Furthermore, *EBV‐miR‐BART18‐3p* KD reduced the mRNA levels of LDHA but not HIF‐1*α* (*P* LDHA: *P* = 0.0287, HIF‐1*α*: *P* = 0.3296) (Figure 4B). In contrast, *EBV‐miR‐BART18‐3p* OEX significantly increased the protein levels of both LDHA and HIF‐1*α* and reduced the protein levels of the HIF‐1*α* inhibitor SIRT1, with the opposite effect for *EBV‐miR‐BART18‐3p* KD (Figure 4C,D). These results are consistent with the possibility that *EBV‐miR‐BART18‐3p*‐dependent LDHA mRNA upregulation may be mediated by increased HIF‐1*α* protein levels. To verify this possibility, we performed chromatin immunoprecipitation (ChIP) assays in hypoxic SW480 cells, which showed that HIF‐1*α* directly binds to the HRE of the LDHA promoter region, and that *EBV‐miR‐BART18‐3p* KD leads to reduced binding affinity between HIF‐1*α* and LDHA (*P* < 0.01) (Figure 4E).

Given that *EBV‐miR‐BART18‐3p* does not directly regulate HIF‐1*α* mRNA expression but activates its protein expression, we considered the possibility that *EBV‐miR‐BART18‐3p* may target up‐stream inhibitors of HIF‐1*α*. Existing databases (miRbase, miRanda, and TargetScan) cannot predict the target genes of virus‐encoded miRNAs; thus, we evaluated the promoter regions of HIF‐1*α* inhibitor genes and identified eight predicted targets (SIRT1, CBP, FIH, PCAF, PHD1, PHD2, PHD3, and VHL) for *EBV‐miR‐BART18‐3p*. In luciferase reporter assays, *EBV‐miR‐BART18‐3p* mimic reduced the activity of a SIRT1 WT 3′‐UTR construct, but not a mutant 3′‐UTR construct, thus confirming that *EBV‐miR‐BART18‐3p* directly targets the SIRT1 3’UTR (Figure 4F,G). Effects of *EBV‐miR‐BART18‐3p* on the other seven predicted targets could not be confirmed (Figure S7, Supporting Information), suggesting that *EBV‐miR‐BART18‐3p*‐mediated repression is specific for SIRT1. In addition, *EBV‐miR‐BART18‐3p* OEX dramatically reduced SIRT1 mRNA levels, while KD increased SIRT1 mRNA levels in hypoxic CRC cells (DLD‐1: *P* = 0.0047; SW480: *P* = 0.0366) (Figure 4H). To determine whether SIRT1 can regulate HIF‐1*α* binding to the LDHA promoter, we performed a ChIP assay in hypoxic SW480 cells with SIRT1 OEX or NC. SIRT1 OEX reduced the binding affinity between HIF‐1*α* and LDHA (Figure 4I, *P* < 0.001), we confirmed the expression of LDHA in hypoxic DLD‐1 and SW480 cells with SIRT1 overexpression by qRT‐PCR assay. The results showed that SIRT1 overexpression reduced the LDHA mRNA levels in hypoxic DLD‐1 and SW480 cells (DLD‐1, *P* = 0.0135; SW480, *P* = 0.0081) (Figure S8, Supporting Information), thus indicating that SIRT1 may function as an intermediate between *EBV‐miR‐BART18‐3p* and HIF‐1*α*/LDHA in CRC.

LDHA is known to catalyze the conversion from lactate to pyruvate during glycolytic metabolism,^[^ ^38^, ^39^, ^40^ ^]^ and several studies have reported that the lactate levels are abnormally elevated and positively correlated with poor prognosis in CRC patients.^[^ ^41^, ^42^, ^43^ ^]^ Therefore, we evaluated the effect of *EBV‐miR‐BART18‐3p* and LDHA expression on intracellular and extracellular lactate and pyruvate levels in CRC cells. As shown in Figure 4J, lactate levels were significantly downregulated in hypoxic SW480 cells and their cell culture medium after KD of either *EBV‐miR‐BART18‐3p* or LDHA expression (intracellular, *P* < 0.01 in *EBV‐miR‐BART18‐3p* group, *P* < 0.01 in LDHA group; extracellular, *P* < 0.05 in *EBV‐miR‐BART18‐3p* group, *P* < 0.001 in LDHA group); in contrast, the intracellular and extracellular pyruvate levels were not affected by *EBV‐miR‐BART18‐3p* or LDHA KD (*P* > 0.05, Figure S9, Supporting Information). Studies have shown that CRC cells can metabolize abnormally elevated lactate levels into acetate, which provides a substrate for the production of acetyl‐CoA and other biological metabolic processes.^[^ ^44^ ^]^ To determine whether *EBV‐miR‐BART18‐3p*‐mediated increase in lactate levels is associated with the activation of LDHA‐dependent glycolytic metabolism, we added ^13^C‐labeled lactate to the medium of hypoxic NC or *EBV‐miR‐BART18‐3p* KD SW480 cells and cultured the cells for 10 h. As shown in Figure 4K, a dynamic flux was observed in which ^13^C‐labeled lactate in extracellular medium was transferred intracellularly and metabolized to pyruvate and acetate; the ^13^C‐labeled acetate was transferred to the nucleus and metabolized to acetyl‐CoA; and then the ^13^C‐labeled acetyl‐CoA was transported back to the cytoplasm. Notably, this metabolic process was significantly attenuated in *EBV‐miR‐BART18‐3p* KD cells. Taken together, our results confirm that *EBV‐miR‐BART18‐3p* enhances acetyl‐CoA production via the SIRT1/HIF‐1*α*/LDHA axis.

### 2.5 *EBV‐miR‐BART18‐3p* Promotes Histone Acetylation and Enhances de novo Lipogenesis

**Figure 5:**
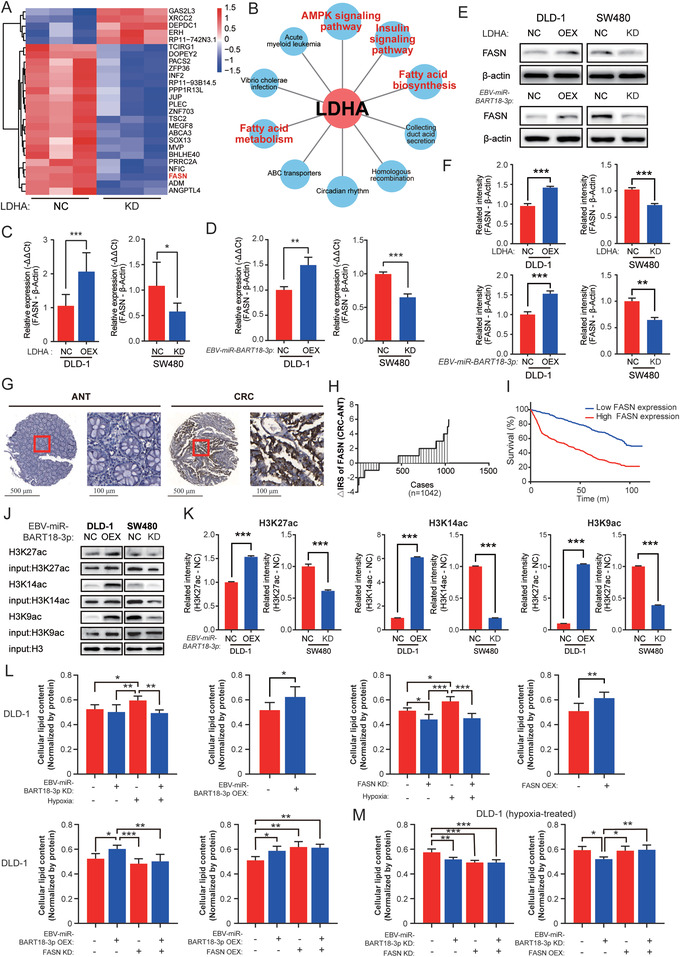
*EBV‐miR‐BART18‐3p* promotes histone acetylation and enhances de novo lipogenesis. A) Heatmap showing differentially expressed mRNAs in hypoxia‐treated, LDHA NC or KD SW480 cells identified by RNA‐seq (*n* = 3/group). B) The top 10 KEGG enrichment pathways of differentially expressed mRNA. The red character‐labeled pathways involve FASN. C) FASN expression levels in hypoxia‐treated CRC cells (LDHA NC vs KD; or LDHA NC vs OEX). Data are presented as −ΔΔCt (*t*‐test, *n* = 3/group). D) FASN expression in hypoxia‐treated CRC cell lines (*EBV‐miR‐BART18‐3p* NC vs KD; or *EBV‐miR‐BART18‐3p* NC vs OEX). Data are presented as −ΔΔCt (*t*‐test, *n* = 3/group). E) Western blot of FASN protein levels in hypoxia‐treated CRC cells (LDHA NC vs KD; LDHA NC vs OEX; *EBV‐miR‐BART18‐3p* NC vs KD; and *EBV‐miR‐BART18‐3p* NC vs OEX). F) Quantifications of western blot results (*t*‐test, three technical replicates each). G) Representative images of FASN expression in TMAs. H) Immune reactivity scores (IRS) of FASN expression in TMAs. I) Kaplan–Meier curves showing the overall survival of CRC patients according to tumoral FASN levels in TMAs (log‐rank test). J) DNA pull‐down and western blot assays showing protein levels of H3K9ac, H3K14ac, H3K27ac in hypoxia‐treated CRC cells (*EBV‐miR‐BART18‐3p* NC vs KD; or *EBV‐miR‐BART18‐3p* NC vs OEX). H3 was used as the loading control. K) Quantifications of western blot results (*t*‐test, three technical replicates each). L) Quantification of the lipid contents in DLD‐1 cells (one‐way ANOVA, *n* = 3/group). M) Lipid contents were measured and quantified in hypoxia‐treated DLD‐1 cells (one‐way ANOVA, *n* = 3/group). n.s.: no significance, *P < 0.05, **P < 0.01, ***P < 0.001.

To further elucidate the downstream signaling mechanisms of LDHA in *EBV‐miR‐BART18‐3p*‐dependent CRC development, we performed RNA‐seq analysis of hypoxic LDHA KD and NC SW480 cells. A total of 26 genes were differentially expressed, of which 21 genes were upregulated and five were downregulated (**Figure** **5**A, fold change > 1.5 and *P* < 0.05). Pathway enrichment analysis for these genes revealed multiple tumor‐associated metabolic pathways (Figure 5B). Within the top 10 enriched pathways, fatty acid synthase (FASN) was identified as a key gene regulated by both LDHA and *EBV‐miR‐BART18‐3p* (Figure 5C,D and Table S10, Supporting Information). Consistent results were also observed for FASN at the protein level (Figure 5E,F). Finally, FASN expression levels were evaluated within the TMA cohort. Results showed that FASN was highly expressed in CRC, and that CRC patients with higher FASN expression in tumors had shorter overall survival when classified by TNM stage (Figure 5G–I; Figure S10 and Tables S11 and S12, Supporting Information).

FASN can use acetyl‐CoA as a substrate for lipid synthesis,^[^ ^45^, ^46^, ^47^ ^]^ and enhanced histone acetylation levels at the FASN promoter region increase de novo lipid synthesis to promote tumor cell survival.^[^ ^48^ ^]^ Because *EBV‐miR‐BART18‐3p* expression mediates nuclear acetyl‐CoA production (Figure 4K), we speculated that elevated LDHA may activate de novo lipogenesis through acetyl‐CoA‐mediated acetylation of histones in the FASN promoter region. To support this possibility, we performed DNA pull‐down and Western blot assays. The results indicate that *EBV‐miR‐BART18‐3p* expression positively regulates the acetylation of histones in the FASN promoter region, including H3K9, H3K14, and H3K27 in hypoxic DLD‐1 and SW480 cells (Figure 5J,K). To analysis the effects of *EBV‐miR‐BART18‐3*p overexpression or knockdown on total histone acetylation, we performed additional western blot analyses of total histone under the same conditions. Results showed that total H3 acetylation was not affected by *EBV‐miR‐BART18‐3*p overexpression or knockdown (Figure S11, Supporting Information). These results suggested that *EBV‐miR‐BART18‐3p* overexpression or knockdown only affected H3 acetylation bound to the FASN promoter region in hypoxic CRC cells.

As additional verification, we used hyperspectral femtosecond stimulated Raman scattering (SRS) microscopy to detect the lipid contents in CRC cells. The ratios of the Raman signals at 2850 (S2850, reflecting the lipid content) and 2928 cm^−1^ (S2928, reflecting the protein contents) were markedly increased in CRC cells (DLD‐1 and SW480) after *EBV‐miR‐BART18‐3p* OEX, FASN OEX, or hypoxic treatment, whereas this effect was abrogated by *EBV‐miR‐BART18‐3p* KD or FASN KD (Figure 5L,M and Figure S12, Supporting Information). These results indicated that *EBV‐miR‐BART18‐3p* and FASN expression could impel CRC cell lipogenesis, which was further confirmed by the results of triglyceride quantification (Figures S13 and S14, Supporting Information). In addition, we have examined the effect of FASN expression on lipid droplet using immunofluorescence assay. BODIPY was a lipophilic fluorescent probe that could be used to label the neutral lipid content of cells, especially those localized to lipid droplets. The results showed that FASN overexpression dramatically increased lipid droplets levels, while knockdown decreased lipid droplets levels in hypoxic CRC cells (Figure S15, Supporting Information). Collectively, our results indicate that increased acetyl‐CoA catalyzes acetylation of histones in the FASN promoter region, which facilitates de novo lipogenesis in CRC cells. Therefore, these findings provide a mechanism for accelerating CRC development via increased *EBV‐miR‐BART18‐3p* expression.

### 2.6 Inhibition of *EBV‐miR‐BART18‐3p* Ameliorates Prognosis in CRC PDX Model Mice

**Figure 6:**
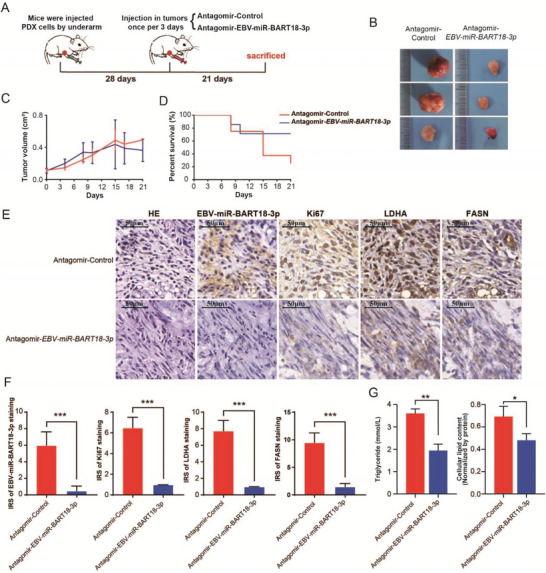
Inhibition of *EBV‐miR‐BART18‐3p* ameliorates CRC progression in a PDX mouse model. A) Schematic diagram of the PDX‐antagomir experimental design. B) Representative tumor images in the PDX‐antagomir model. C) Tumor volumes after intratumoral antagomir injection. Error bar: SEM. D) Survival curves of mice after intratumoral antagomir injection. E) Representative images of PDX tumor serial sections subjected to H&E, *EBV‐miR‐BART18‐3p*, Ki67, LDHA, and FASN staining. F) Quantification of PDX tumor section staining (*t*‐test, *n* = 7/group). G) Lipid and triglyceride contents of PDX tumor tissues (*t*‐test, *n* = 7/group). n.s.: no significance, *P < 0.05, **P < 0.01, ***P < 0.001.

To evaluate the therapeutic potential of decreasing *EBV‐miR‐BART18‐3p* levels in vivo, we established a CRC patient‐derived xenograft (PDX)‐antagomir model. At 28 d after PDX cell injection, we divided seven pairs of xenografted mice into two groups and intratumorally injected antagomir‐*EBV‐miR‐BART18‐3p* or antagomir‐NC once every 3 d for an additional 21 d (**Figure** **6**A). As shown in Figure 6B,C, *EBV‐miR‐BART18‐3p* antagomir significantly slowed tumor growth, especially later in the experiment (12–21 d after antagomir injection, *P* = 0.0171). Furthermore, *EBV‐miR‐BART18‐3p* antagomir improved the overall survival of mice (Figure 6D) but had little effect on body weight (Figure S16, Supporting Information). To corroborate that antagomir‐*EBV‐miR‐BART18‐3p* injection decreases tumoral *EBV‐miR‐BART18‐3p* expression levels, we performed immunohistochemical staining of PDX tumor tissue serial sections. The results confirm that antagomir‐*EBV‐miR‐BART18‐3p* decreased *EBV‐miR‐BART18‐3p* expression and also demonstrate that antagomir‐*EBV‐miR‐BART18‐3p* decreased the levels of LDHA and FASN, as well as Ki67 (a recognized tumor malignant phenotype marker^[^ ^49^ ^]^) in tumor tissues (Figure 6E,F). To evaluate the effect of antagomir‐*EBV‐miR‐BART18‐3p* on de novo lipogenesis, we prepared a single cell suspension by mechanically disaggregating xenograft tumors. Consistent with the in vitro findings, the triglyceride contents were significantly decreased in the antagomir‐*EBV‐miR‐BART18‐3p* injection group compared to the antagomir‐NC group (Figure 6G). Collectively, these data provide evidence that *EBV‐miR‐BART18‐3p* promotes CRC development by activating *de novo* lipogenesis in CRC cells through the SIRT1/HIF‐1*α*/LDHA/FASN axis and suggest that *EBV‐miR‐BART18‐3p* may serve as a novel therapeutic target for CRC treatment.

## 3 Discussion

Viral infection is potential risk factor for the development and progression of colorectal cancer;^[^^]^ however, the link between virus infections and CRC has not been well characterized. In the current study, we demonstrated that an EBV‐encoded miRNA, *EBV‐miR‐BART18‐3p*, significantly promotes tumor growth and facilitates de novo lipogenesis in CRC by regulating the SIRT1/HIF1*α*/LDHA/FASN axis. Our study strongly links *EBV‐miR‐BART18‐3p* to de novo lipogenesis and CRC carcinogenesis.

EBV‐encoded miRNAs have inspired much interest for their rich abundance and potential functions.^[^ ^50^ ^]^ A recent study reported that *EBV‐miR‐BART7‐3p* promotes epithelial‐to‐mesenchymal transition and nasopharyngeal carcinoma metastasis by targeting PTEN.^[^ ^54^ ^]^ *EBV‐miR‐BART2‐5p* also has been shown to target RND3 to activate Rho signaling and promote nasopharyngeal carcinoma metastasis.^[^ ^52^ ^]^ Furthermore, *EBV‐miR‐BART17‐5p* promotes gastric carcinoma cell migration and anchorage‐independent growth by targeting KLF2.^[^ ^55^ ^]^ Despite these demonstrated roles of EBV‐miRNAs in cancer development,^[^ ^14^, ^56^ ^]^ little is known about their roles in CRC. In the present study, we reported that *EBV‐miR‐BART18‐3p* is extensively involved in the progression of CRC and demonstrated a mechanism that explains its activity.

The tumor microenvironment is characterized by dynamic gradients of oxygen diffusion and consumption.^[^ ^57^ ^]^ Hypoxia is an important feature of tumor microenvironment and is a common character in approximately half of all soild tumors.^[^ ^58^ ^]^ Hypoxia is an important feature of tumor microenvironment and is a common character in approximately half of all solid tumors. In addition, hypoxia can not only induce miRNA expression in many tumor cells but can also regulate normal cells by encapsulation of miRNA. And these miRNAs are associated with enhanced angiogenesis. In this way, tumor cells activate multiple survival pathways to complete necessary biological processes. The studies indicate that hypoxia leave a specific mark on miRNA profiles in a variety of tumor cell types, with a critical contribution of the hypoxia‐inducible factor (HIF). Our study establishes that *EBV‐miR‐BART18‐3p* functions as an oncogenic factor in CRC development, increasing under hypoxia. The increased expression of *EBV‐miR‐BART18‐3p* is probably a contribution of hypoxia inducible factor (HIF). Hypoxia was a common mechanism of HIF activation in cancer. Hypoxia regulated HIF‐1 at the level of protein stability by inhibiting its ubiquitin‐mediated degradation.^[^ ^59^ ^]^ Under hypoxic conditions in cell culture, HIF‐1*α* mRNA did not change, but HIF‐1*α* protein levels increased.^[^ ^60^, ^61^, ^62^ ^]^ However, our results were consistent with previous studies in that no changes in the mRNA level of HIF‐1*α* were observed.^[^ ^59^ ^]^ But *EBV‐miR‐BART18‐3p* overexpression significantly increased HIF‐1*α* protein levels but not mRNA levels in hypoxic CRC cells.

Previously, the underlying mechanism of EBV‐miRNAs in cancer development has been shown to involve direct targeting/inhibition of tumor suppressors. For example, MiR‐BART3* has been shown to target DICE1[63], and *miR‐BART1* has been shown to target PTEN[64], both of which are well‐known tumor suppressors. However, none of these interactions have been demonstrated in CRC cells. Using WGCNA, we identified an *EBV‐miR‐BART18‐3p*–mRNA network involved in CRC development. In contrast to the latter reports, we found that expression of LDHA and *EBV‐miR‐BART18‐3p* were synchronously increased in CRC tissues. LDHA has been shown to regulate by HIF‐1*α* and c‐myc,^[^ ^65^, ^66^ ^]^ and we demonstrate that HIF‐1*α* mediates the regulatory effect of *EBV‐miR‐BART18‐3p* on LDHA in CRC. In addition, our results suggest that HIF‐1*α* mRNA is not a direct target of *EBV‐miR‐BART18‐3p*, given that its protein levels, but not its mRNA expression levels, were altered following *EBV‐miR‐BART18‐3p* OEX or KD in CRC cells. Therefore, we hypothesized that *EBV‐miR‐BART18‐3p* may directly target an inhibitor of HIF‐1*α* to increase HIF‐1*α* expression. Consistently, our results showed that OEX of *EBV‐miR‐BART18‐3p* decreases SIRT1 expression, subsequently increasing HIF‐1*α* and LDHA expression in CRC cells. SIRT1 is a well‐established inhibitor of HIF‐1*α* ^[^ ^67^ ^]^ that functions as an NAD^+^‐dependent protein deacetylase and exerts complex functions in cellular metabolism by deacetylating target proteins in different tissues.^[^ ^68^, ^69^, ^70^ ^]^ Downregulation of SIRT1 has been reported to promote the acetylation and activation of HIF‐1*α* and further activate glycolysis in a positive feedback loop during hypoxia.^[^ ^67^ ^]^ Therefore, our findings suggest that *EBV‐miR‐BART18‐3p* is an upstream driver of the SIRT1/HIF‐1*α*/LDHA pathway.

Malignant cells exhibit abnormal metabolism and become dependent on de novo lipogenesis to survive after cellular stress.^[^ ^71^ ^]^ LDHA catalyzes the conversion of l‐lactate and NAD to pyruvate and NADH, and CRC cells exhibiting high aerobic glycolytic activity contribute to angiogenesis and resistant phenotypes.^[^ ^72^ ^]^ In malignant cells, metabolic substrates produced by glycolysis are funneled into other metabolic pathway, such as de novo lipogenesis, which is indispensable for rapid cancer cell growth.^[^ ^21^, ^71^ ^]^ Multiple studies have focused on the dysregulation of lipid synthesis as a mechanism of preventing growth and survival of cancer cells,^[^ ^73^, ^74^, ^75^ ^]^ especially including the contribution of lipogenic enzymes, with FASN catalyzing the terminal steps in *de novo* biogenesis of fatty acids in cancer pathogenesis.^[^ ^47^ ^]^ In this study, we identified FASN as a key molecule in *EBV‐miR‐BART18‐3p*‐induced lipogenesis. In addition, consistent with our results, the expression of FASN has been reported to be significantly increased in CRC tissues and to be associated with the development of CRC and liver metastasis.^[^ ^78^ ^]^ In addition, inhibition of de novo lipogenesis has been recognized as a promising therapeutic target in CRC treatment.^[^ ^79^ ^]^ In our study, the increased acetyl‐CoA levels induced by the *EBV‐miR‐BART18‐3p* axis enhanced the acetylation levels of histones H3K9, H3K14, and H3K27, which upregulated FASN expression and increased *de novo* lipogenesis. Enhanced histone acetylation levels at the promoter regions of FASN have been reported to promote FASN expression and increase de novo lipid synthesis to promote tumor cell survival,^[^ ^46^ ^]^ thus supporting its role in *EBV‐miR‐BART18‐3p*‐dependent CRC progression.

In conclusion, our study establishes that *EBV‐miR‐BART18‐3p* functions as an oncogenic factor in CRC development by upregulating LDHA‐mediated metabolic processes and the FASN‐mediated de novo lipogenesis pathway. We further ascertained the therapeutic value of *EBV‐miR‐BART18‐3p* in the PDX mouse model. These results serve to advance the current understanding of the mechanisms by which EBV infection‐induced upregulation of exogenous miRNAs may be a risk factor for CRC and suggest that *EBV‐miR‐BART18‐3p* might serve as a potential diagnostic marker and therapeutic target for CRC.

## 4 Conclusion

In summary, we reported that *EBV‐miR‐BART18‐3p* contributed to and promoted CRC metastasis during EB virus injection via an altered lipogenesis pathway. Therefore, the maintenance of homeostasis of lipogenesis is important to reduce the EB virus injection–associated CRC aggravation and metastasis to the liver or lung.

## 5 Experimental Section

### Patients and Specimens

All samples used in this study were obtained with the informed consent of the patients and were in compliance with ethical regulations. The study was approved by the Ethics Committee of Southeast University Affiliated Zhongda Hospital (2014ZDSYLL035.0). Six pairs of CRC tissues (three colon and three rectal carcinomas and corresponding adjacent noncancerous tissues) and six tubulovillous adenoma tissues were subjected to mRNA microarray^[^ ^33^ ^]^ (GEO database, GSE104364) and miRNA microarray^[23]^ (GEO database, GSE72281). The specimens were obtained from patients undergoing surgery at the Jiangsu Tumor Hospital in 2015; detailed information is provided in Table S1 (Supporting Information).^[^ ^23^, ^33^ ^]^

Datasets consisting of 135 CRC cases and 135 controls, and 268 CRC cases and 268 controls were enrolled at the Jiangsu Tumor Hospital and the Affiliated Hospital of the Xuzhou Medical College, respectively, between 2014 and 2017. The combined set included 80% of the cases that were randomly selected as the testing set (consisting of 322 CRC cases and 322 controls) and 20% that was used as the validation set (consisting of 81 CRC cases and 81 controls). Peripheral blood samples were also collected from CRC patients in the two sets, and sex‐ and age‐matched healthy donors were enrolled from the Nanjing Hospital of Chinese Medicine from 2014 to 2017 as control subjects; each healthy volunteer was confirmed to have no malignant tumors or infectious diseases. For the CRC patients, the tumor stage was defined according to the criteria of the sixth edition of the American Joint Committee on Cancer (AJCC). Detailed information of CRC patients is provided in Table S3 (Supporting Information).

The CRC and matched nontumoral tissues (5 cm from the tumoral margins) from two independent cohorts were subjected to TMA construction. A total of 376 patients were recruited from the Affiliated Hospital of Xuzhou Medical College between 2007 and 2011, and 702 patients were recruited from the Jiangsu Tumor Hospital between 2007 and 2011 for the TMA. Among these cases, 80% were randomly selected from the combined cohort as the testing set (consisting of 862 CRC cases and 862 corresponding controls), and the other 20% was used as the validation set (consisting of 216 CRC cases and 216 corresponding controls). All patients were followed up by a trained clinical specialist through in‐person or family visits from the time of diagnosis to the date of death or final follow‐up. The maximum follow‐up time was 112.7 months, and the median survival time (MST) was 67.1 months. Detailed information on these patients is provided in Table S4 (Supporting Information).

### miRNA Expression Microarrays

Total RNA was isolated from tissues using TRIzol Reagent (Invitrogen, Carlsbad, CA) according to the manufacturer's instructions. The miRNA microarrays were performed using the miRCURY LNA Array System (V3.0) (Exiqon, Vedbaek, Denmark) by a contract service at Shanghai Kangcheng Technology (Shanghai, China). Raw data were subjected to background subtraction and normalization with the *limma R*‐package. Discriminant miRNAs and differences between groups were analyzed using the Bayes moderated *t*‐test (*limma*) with the Benjamini–Hochberg false discovery rate (FDR) at *P* < 0.05, unless otherwise specified. A two‐fold change cut‐off and *P* value < 0.05 were applied to select the up‐ and downregulated miRNAs. Normalized and raw expression data were deposited in the Gene Expression Omnibus at the National Center for Biotechnology Information.

### RNA‐Seq Analysis

RNA‐Seq experiments were performed by Novogene (Beijing, China). Briefly, an mRNA‐seq library was prepared for sequencing using standard Illumina protocols. Total RNA from SW480 control (NC) and LDHA knockdown (KD) cells was isolated using TRIzol reagent (Invitrogen, Carlsbad, CA) and treated with RNase‐free DNase I (New England Biolabs, Ipswich, MA) to remove any contaminating genomic DNA. The mRNA extraction was performed with Dynabeads oligo (dT) (Invitrogen Dynal, Oslo, Norway). Double‐stranded complementary DNA was synthesized using Superscript II reverse transcriptase (Invitrogen, Carlsbad, CA) and random hexamer primers. Next, the cDNA was fragmented by nebulization, and the standard Illumina protocol was followed thereafter to create an mRNA‐seq library. For data analysis, base‐calling was performed using CASAVA. Reads were aligned to the genome using the split read aligner TopHat (v2.0.7) and Bowtie2 using default parameters, and HTSeq was used to estimate abundances. Differential expression analyses between three conditions/groups were performed using the DESeqR package (1.10.1). DESeq provides statistical routines to determine differential expression in digital gene expression data using a model based on the negative binomial distribution. The resulting *P*‐values were adjusted using the Benjamini and Hochberg's approach to control for FDR. Genes with an adjusted *P*‐value < 0.05 identified by DESeq were designated as differentially expressed.

### Animals

Five‐week‐old nude mice and NOD‐Prkdc^em26Cd52^Il2rg^em26Cd22^/Nju (NCG) mice were purchased from the Model Animal Research Center of Nanjing University (China). All animals were housed at 24 ± 2 °C, with 50 ± 1% relative humidity and a 12/12 h light/dark cycle and were given water and a basal diet ad libitum. All animal studies (including the procedure for mouse euthanasia) were done in compliance with the regulations and guidelines of the Institutional Animal Care Committee of Southeast University and were conducted according to the guidelines of the Association for Assessment and Accreditation of Laboratory Animal Care.

For nude mice xenograft tumor models, SW480 or RKO NC, *miR‐EBV‐BART18‐3p* KO cells and DLD‐1 or SW620 NC, *miR‐EBV‐BART18‐3p* OEX cells (1 × 10^6^ cells/100 µL PBS) were subcutaneously injected into the flanks of nude mice (six mice/group). Bidimensional tumor measurements were taken with vernier calipers. Tumor volumes were calculated according to the following formula: Volume (mm^3^) = 0.5 × length × width^2^. The mice were sacrificed four weeks after xenograft injection, and tumor tissues were harvested for immunostaining analysis.

For the PDX mouse model, fresh human CRC tissues from patients were collected, washed twice with cold PBS containing penicillin (500 U mL^−1^) and streptomycin (500 µg mL^−1^), sectioned into small fragments, and transplanted subcutaneously into the flanks of NCG mice. When the tumor volumes reached 250 mm^3^, the xenografts were resected and reimplanted into new NCG mice. Successfully xenografted CRC tumor models were obtained after three passages. Written informed consent was obtained from each patient, and detailed patient information is provided in Table S14 (Supporting Information).

For PDX‐antagomir experiments, PDX tumors from each source of PDX mice in passage 4 were disaggregated and then washed twice using PBS containing 500 U mL^−1^ penicillin, 500 µg mL^−1^ streptomycin, 100 mg L^−1^ gentamicin, and 2.5 mg L^−1^ amphotericin B. Next, the tumor mass was digested in culture medium containing 1 mg mL^−1^ type II/IV collagenase and 1 mg mL^−1^ DNase at 37 °C for 1 h with gentle shaking. After filtration through an 80 µm filter (BD Biosciences, San Jose, CA), the cell suspensions were centrifuged at 300 *g* for 5 min at 4 °C. The pellets were resuspended using Dulbecco's Modified Eagle Medium (DMEM), and then nude mice were injected under the arm with 1 × 10^7^ PDX cells. After 28 d, seven pairs of successfully xenografted mice were divided into two groups, and antagomir‐NC or antagomir‐*EBV‐miR‐BART18‐3p* was injected intratumorally once every 3 d. The mice were sacrificed after 21 d.

The rest detailed material and methods are provided in the Supporting Information.

## Conflict of Interest

The authors declare no conflict of interest.

## Supporting information

Supporting information

## Data Availability

The data can be obtaine from the GEO database (GSE104364 and GSE72281).
