# Supplementary material for: Epstein–Barr Virus‐Encoded MicroRNA‐BART18‐3p Promotes Colorectal Cancer Progression by Targeting De Novo Lipogenesis
Source: Adv Sci (Weinh). 2022 Oct 28;9(35):2202116. doi: 10.1002/advs.202202116 (PMC9762317; doi:10.1002/advs.202202116)
Supplement: Supplementary file 1 — Supporting information [file ADVS-9-2202116-s001.pdf]

ADVANCED SCIENCE
Open Access

Supporting Information

for *Adv. Sci.*, DOI 10.1002/advs.202202116

Epstein–Barr Virus-Encoded *MicroRNA-BART18-3p* Promotes Colorectal Cancer Progression by Targeting De Novo Lipogenesis

Qingtao Meng, Hao Sun, Shenshen Wu, Giuseppe Familiari, Michela Relucenti, Michael Aschner, Xiaobo Li and Rui Chen**

Supplementary Figures

Figure. S1 Expression profile of differentially expression microRNAs

(A) Heatmap of differentially expressed miRNAs across ANT, ADE and CRC samples (n = 6/group). **(B)** Venn diagram showing the pattern of significantly altered miRNAs among the 3 types of samples. **(C)** Heatmap showing the levels of the 3 common differentially expressed miRNAs from the overlapping region of the Venn diagrams.

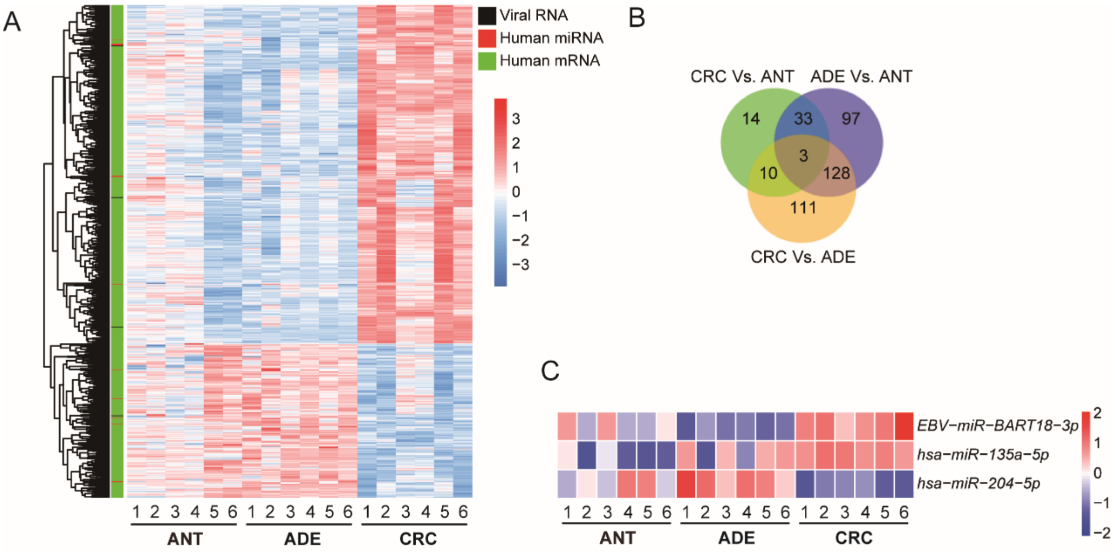

Figure. S2 *EBV-miR-BART18-3p* expression is positively correlated with CRC progression. (A) *EBV-miR-BART18-3p* expression levels in fresh tissues from the testing and validation sets for CRC (CRC) and adjacent noncancerous (ANT) were measured by PCR. Data are presented as −ΔΔCt (***P < 0.001, t-test, 3 technical replicates each). **(B)** Receiver operating characteristic (ROC) curves according to the expression levels of *EBV-miR-BART18-3p*. **(C)** Immune reactive scores of the testing and validation sets for *EBV-miR-BART18-3p* levels in tissue microarrays (TMAs). **(D)** Kaplan–Meier curves of overall survival in the testing and validation sets of CRC patients according to tumoral *EBV-miR-BART18-3p* levels in TMAs (Log-rank test).

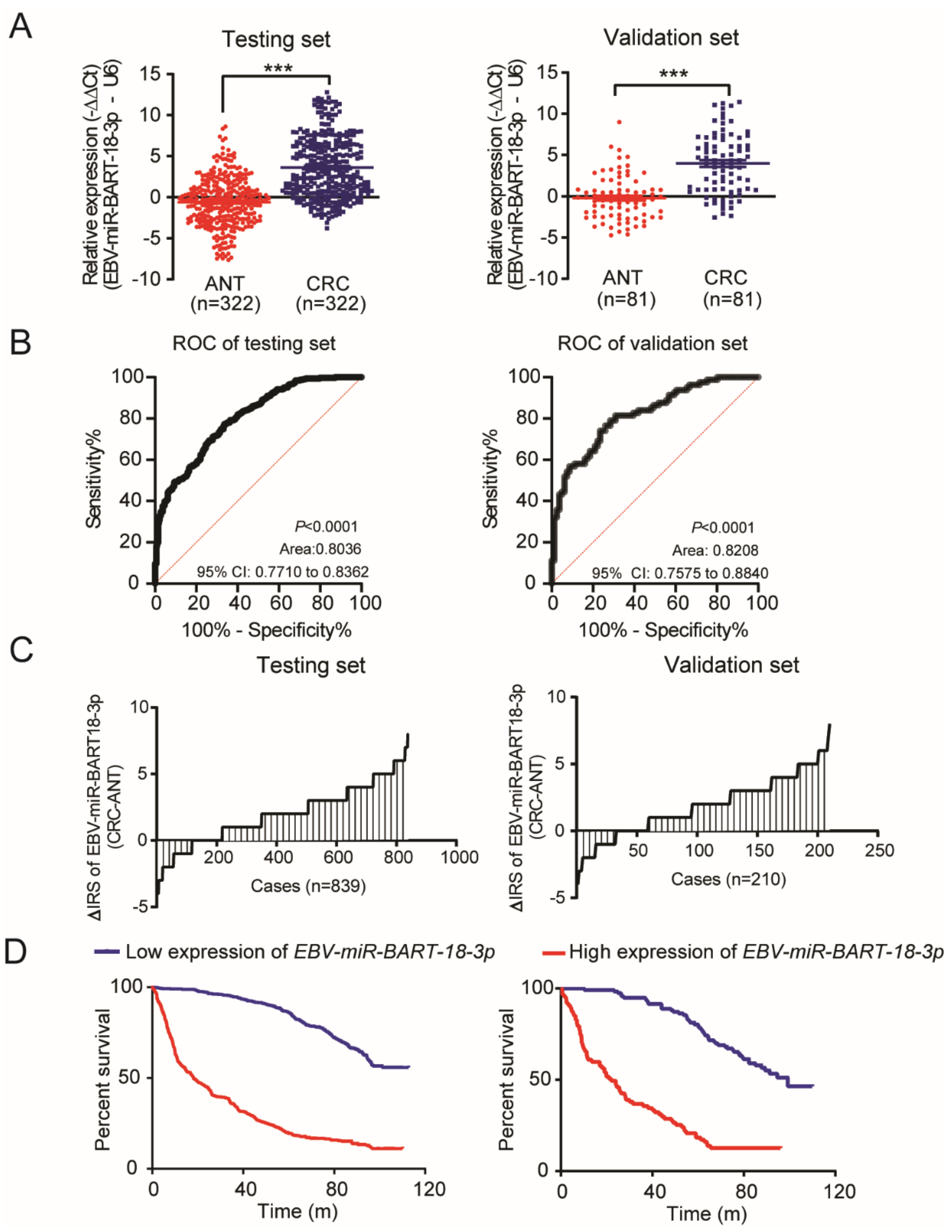
A
Testing set
Relative expression (-ΔΔCt)
(EBV-miR-BART-18-3p - U6)
***
ANT
(n=322)
CRC
(n=322)
Validation set
***
ANT
(n=81)
CRC
(n=81)
B
ROC of testing set
Sensitivity%
P<0.0001
Area:0.8036
95% CI: 0.7710 to 0.8362
100% - Specificity%
ROC of validation set
P<0.0001
Area: 0.8208
95% CI: 0.7575 to 0.8840
C
Testing set
ΔIRS of EBV-miR-BART18-3p
(CRC-ANT)
Cases (n=839)
Validation set
Cases (n=210)
D
Low expression of EBV-miR-BART-18-3p
High expression of EBV-miR-BART-18-3p
Percent survival
Time (m)

Figure. S3 *EBV-miR-BART18-3p* expression levels in the serum of healthy donors and CRC patients at different pathological stages and corresponding ROC curves. (A)T, **(B)** N, **(C)** M (*t*-test, 3 technical replicates each)

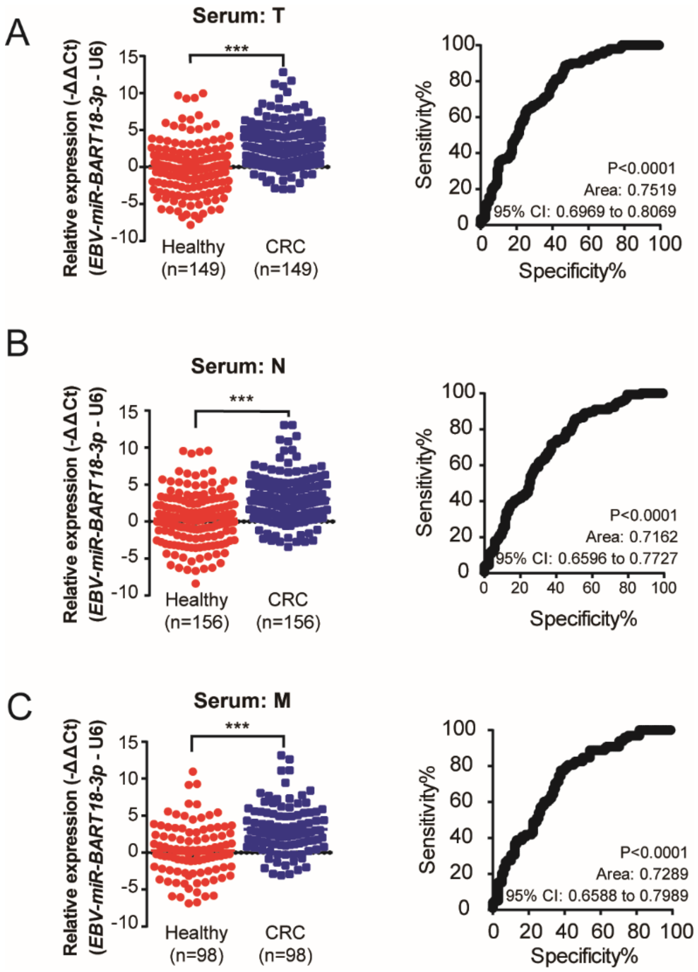

Figure. S4 *EBV-miR-BART18-3p* expression levels in CRC cells and human normal colonic mucosal epithelial (NCM460) cells. Data were obtained by PCR analysis and are presented as −ΔΔCt (n = 3/group).

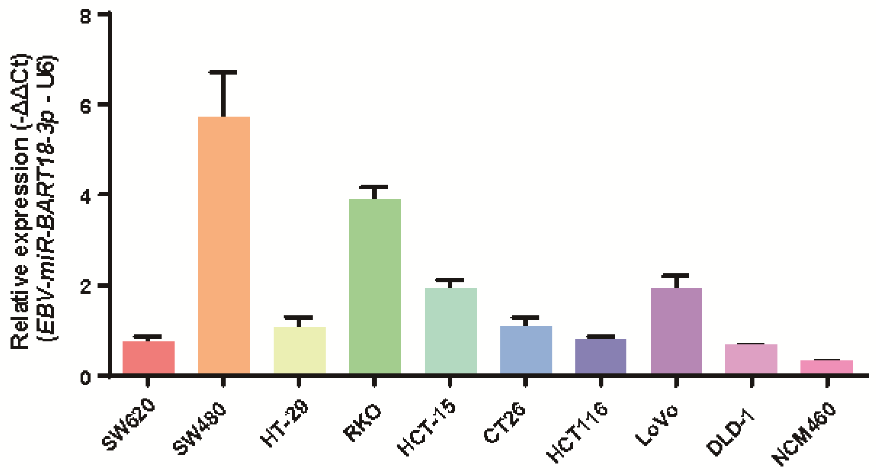

Figure. S5 ***EBV-miR-BART18-3p*** **enhances CRC malignant phenotypes** ***in vitro*** **and** ***in vivo*****. (A)** Migratory and invasive capacities in *EBV-miR-BART18-3p* NC and OEX SW620 cells (*t*-test, n=3/group). **(B)** Migratory and invasive capacities in *EBV-miR-BART18-3p* NC and KD RKO cells (*t*-test, n=3/group). **(C and D)** Tumor volume of DLD-1 and SW480 cell xenografts in mice (*t*-test, n=3/group). **(E and F)** Representative images of SW620 or RKO cell xenografts, and tumor volumes in mice. (**G and H**) Luciferase activity of xenograft, liver and lung tissues from mice following subcutaneous injection of SW620 or RKO cells (*t*-test, n=3/group). $^{*}P < 0.05$ $^{**}P < 0.01$

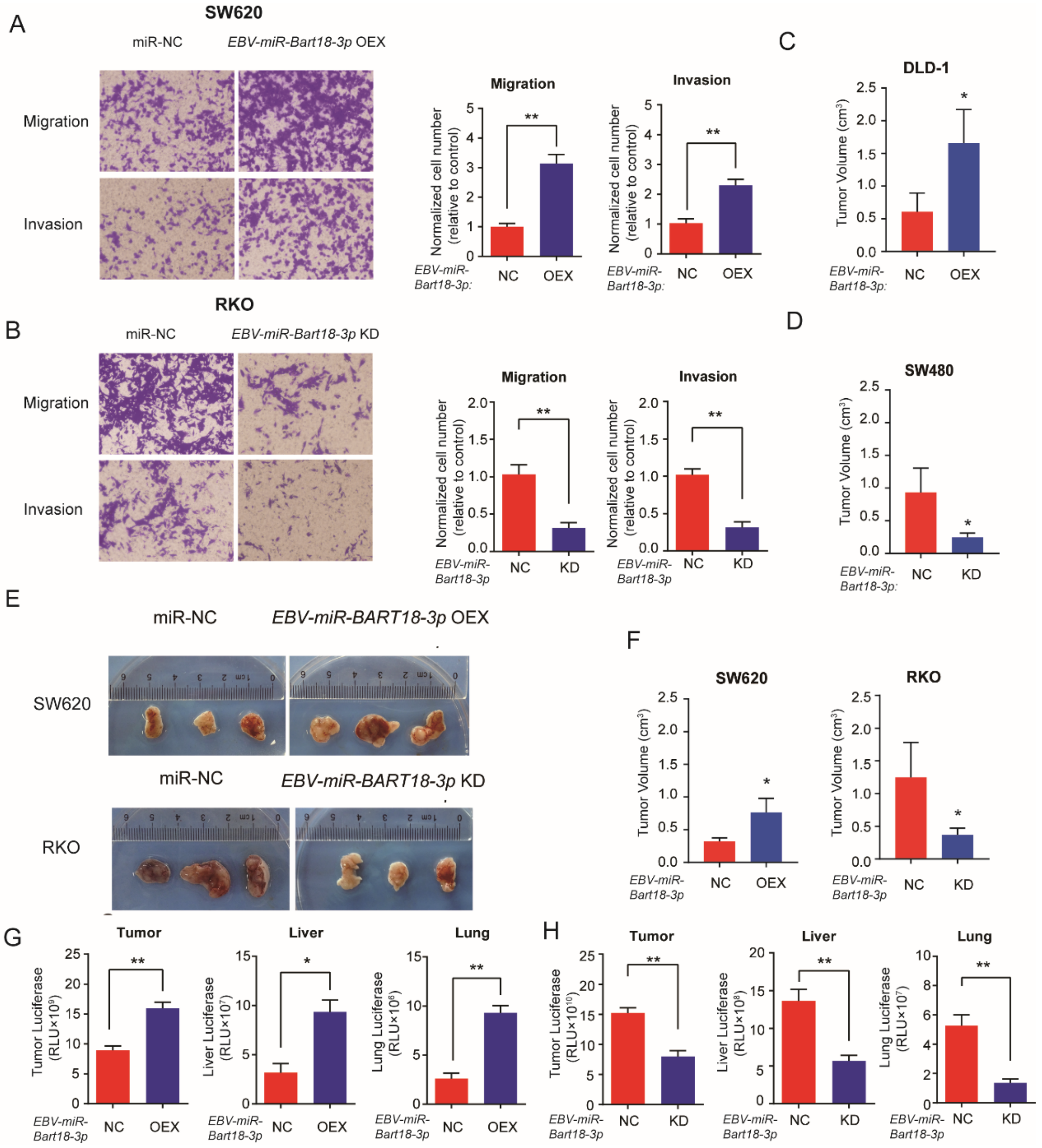

Figure. S6 Venn diagrams of gene number in the original black module and the secondary turquoise module in WGCNA

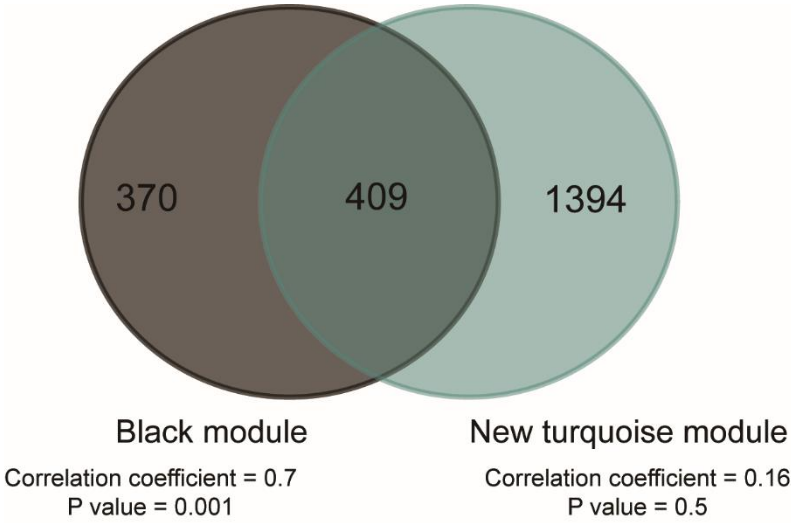

Figure. S7 Schematic and binding abilities of transcription factors to *EBV-miR-BART18-3p*. (A) CBP **(B)** FIH **(C)** PCAF **(D)** PHD1 **(E)** PHD2 **(F)** PHD3 **(G)** VHL (n.s., no significance, *t*-test, n=3/group).

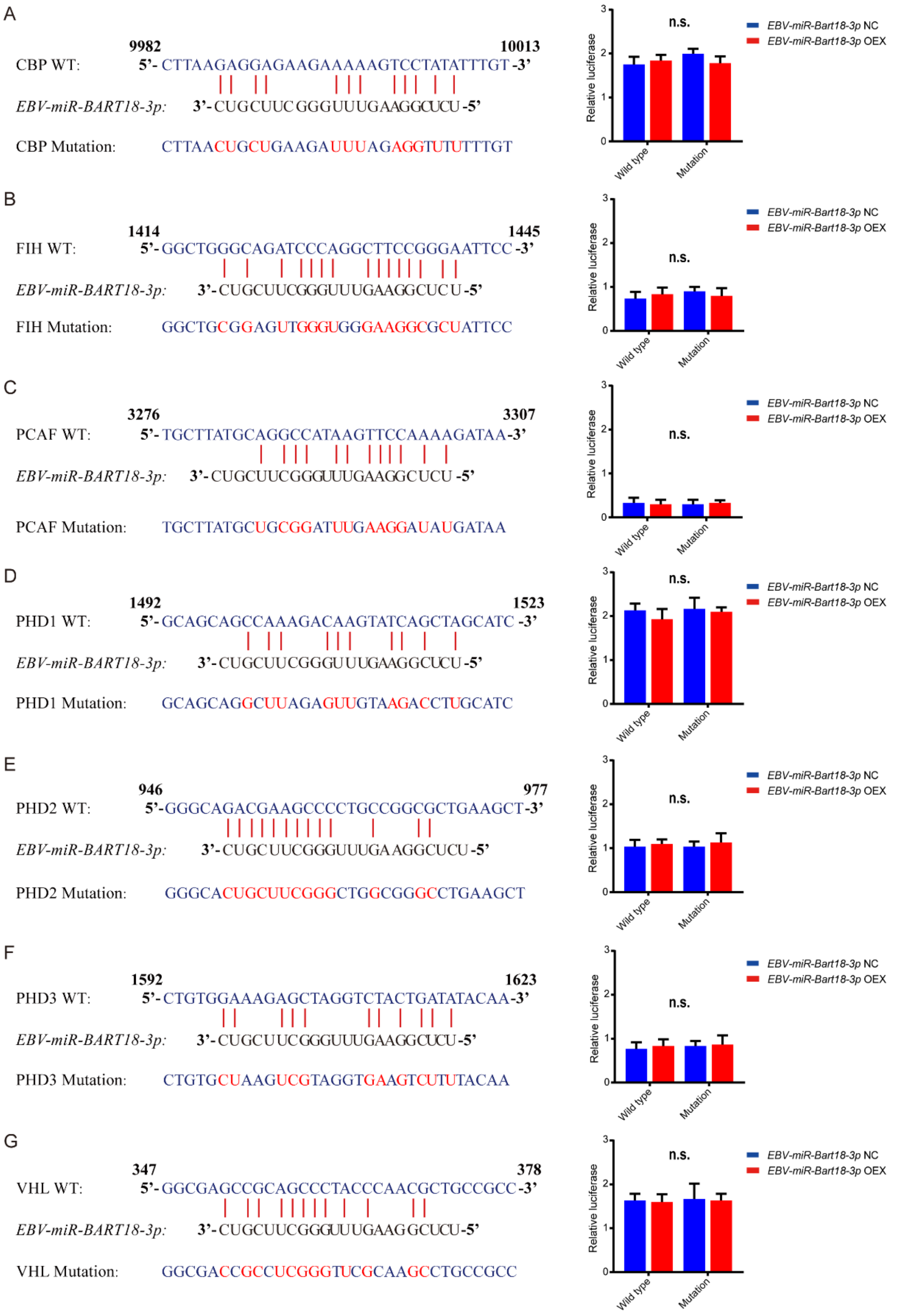

Figure. S8 SIRT1 overexpression suppresses HIF-1alpha binding to the LDHA promoter. The expression of LDHA were detected with SIRT1 overexpression in hypoxic DLD-1 **(A)** and SW480 cells **(B)** by qRT-PCR assay**.** **P* < 0.05 ***P* < 0.01

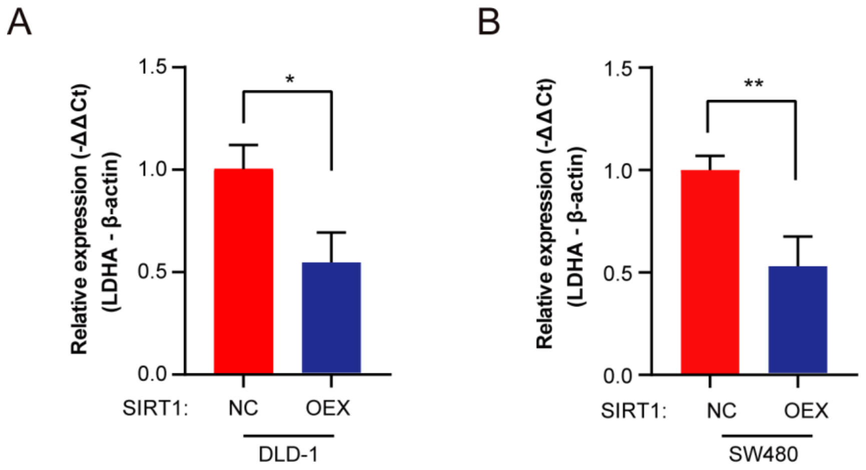

Figure. S9 Intracellular and extracellular pyruvate levels. (n.s. no significance, $P > 0.05$, t-test, 3 technical replicates each).

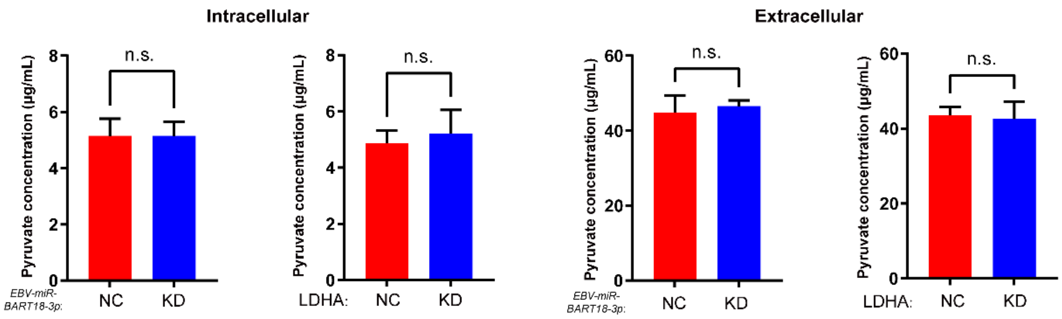

Figure. S10 Immune reactive scores of FASN staining in TMAs, and the overall survival time of CRC patients according to the FASN expression levels. (log-rank test).

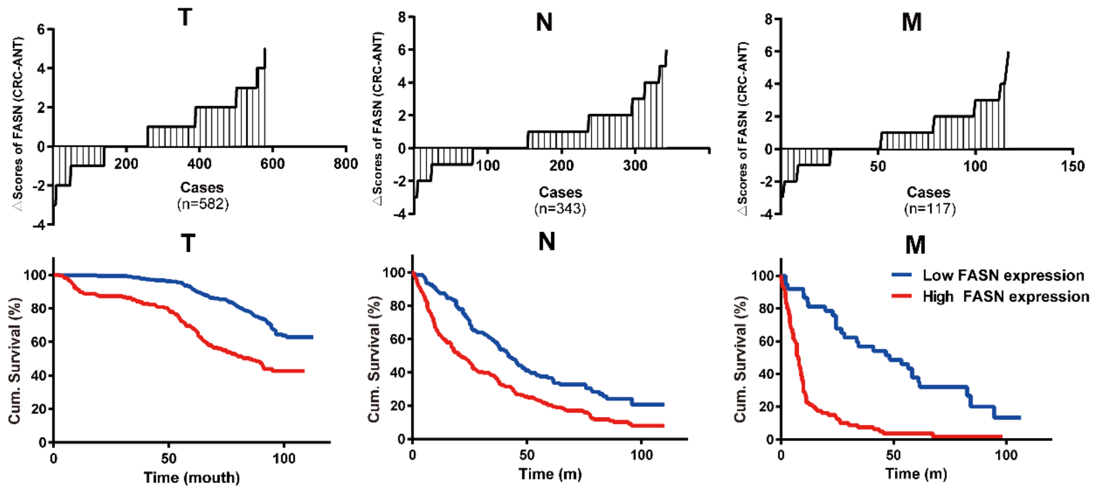

Figure. S11 Total H3 acetylation is not affected by *EBV-miR-BART18-3p* overexpression or knockdown. Western blot assays showed protein levels of H3K9ac, H3K14ac, H3K27ac in hypoxia-treated CRC cells (*EBV-miR-BART18-3p* NC *versus* KD; or *EBV-miR-BART18-3p* NC *versus* OEX). H3 was used as the loading control.

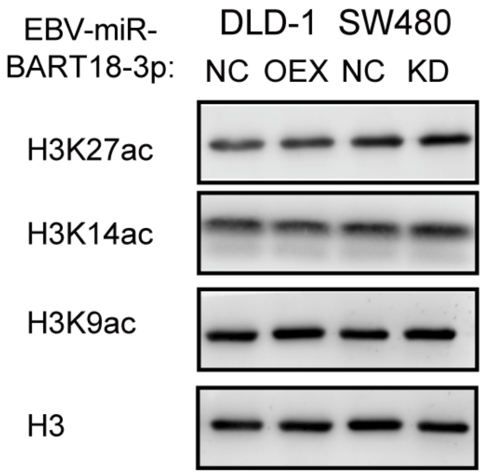

Figure. S12 *EBV-miR-BART18-3p* enhances FASN-mediated lipid synthesis in SW480 cells. (A) lipid contents were measured and quantified in SW480 cells (one-way ANOVA, n=3/group). **(B)** lipid contents were measured and quantified in hypoxia-treated SW480 cells (one-way ANOVA, n=3/group). **P* < 0.05, ***P* < 0.01, ****P* < 0.001.

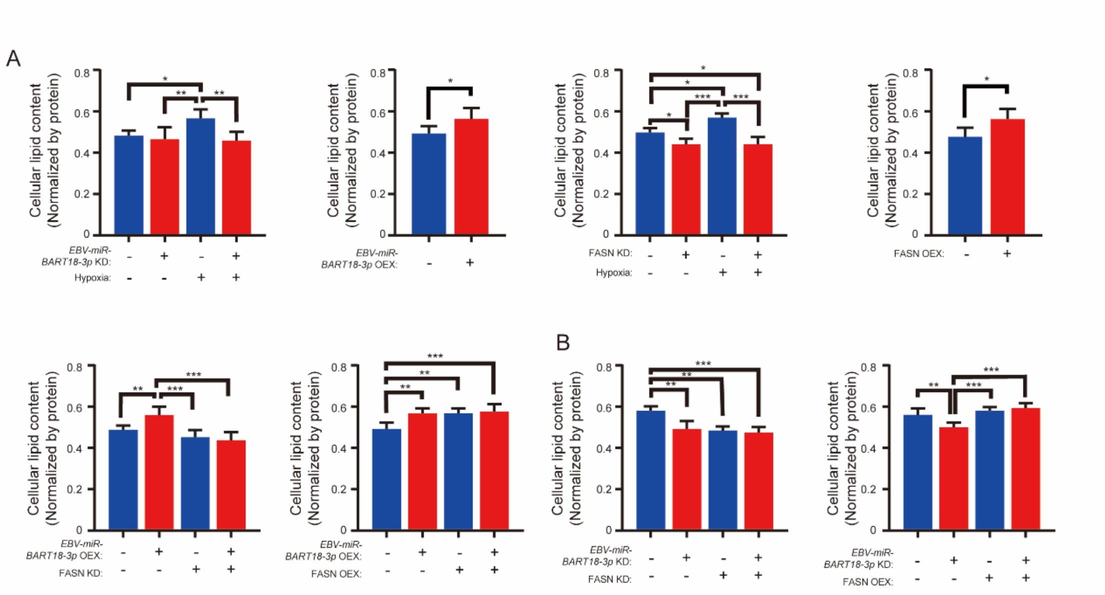

Figure. S13 *EBV-miR-BART18-3p* enhances FASN-mediated triglyceride synthesis in DLD-1 cells. (A) Triglyceride contents were measured and quantified in DLD-1 cells (one-way ANOVA, n=3/group). **(B)** Triglyceride contents were measured and quantified in the indicated hypoxic cultured DLD-1 cells (one-way ANOVA, n=3/group). **P* < 0.05, ***P* < 0.01

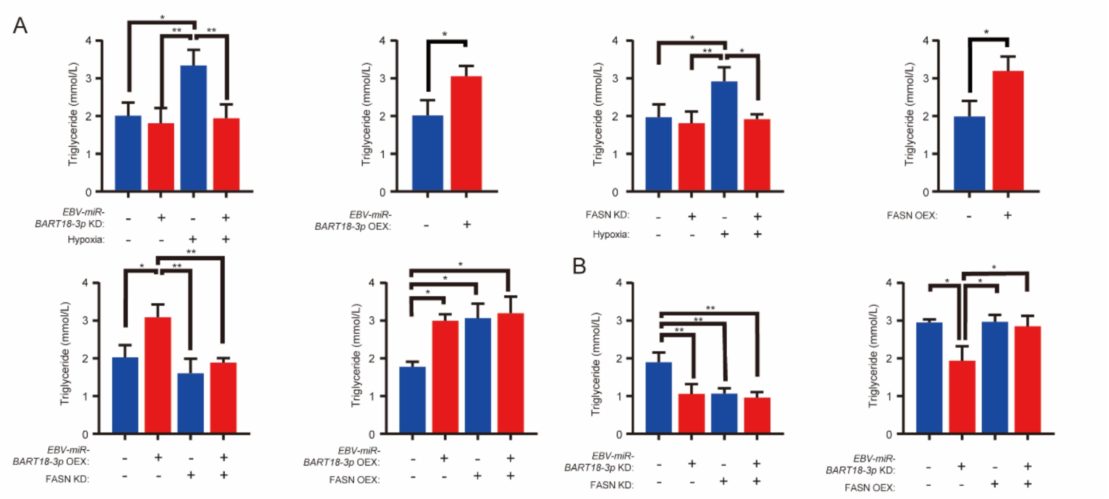

Figure. S14 ***EBV-miR-BART18-3p*** **enhances FASN-mediated triglyceride synthesis in SW480 cells. (A)** Triglyceride contents were measured and quantified in SW480 cells (one-way ANOVA, n=3/group). **(B)** Triglyceride contents were measured and quantified in hypoxia-treated SW480 cells (one-way ANOVA, n=3/group). **P* < 0.05, ***P* < 0.01

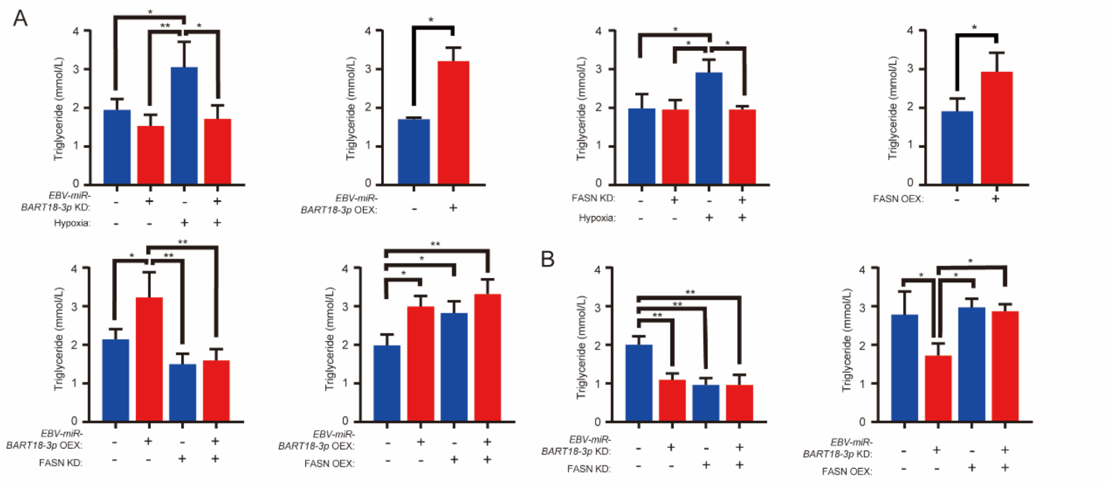

Figure. S15 ***EBV-miR-BART18-3p*** **enhances FASN-mediated lipid droplets in SW480 cells.** Immunofluorescence assays showing the expression levels and intracellular localization of lipid droplets in CRC cells.

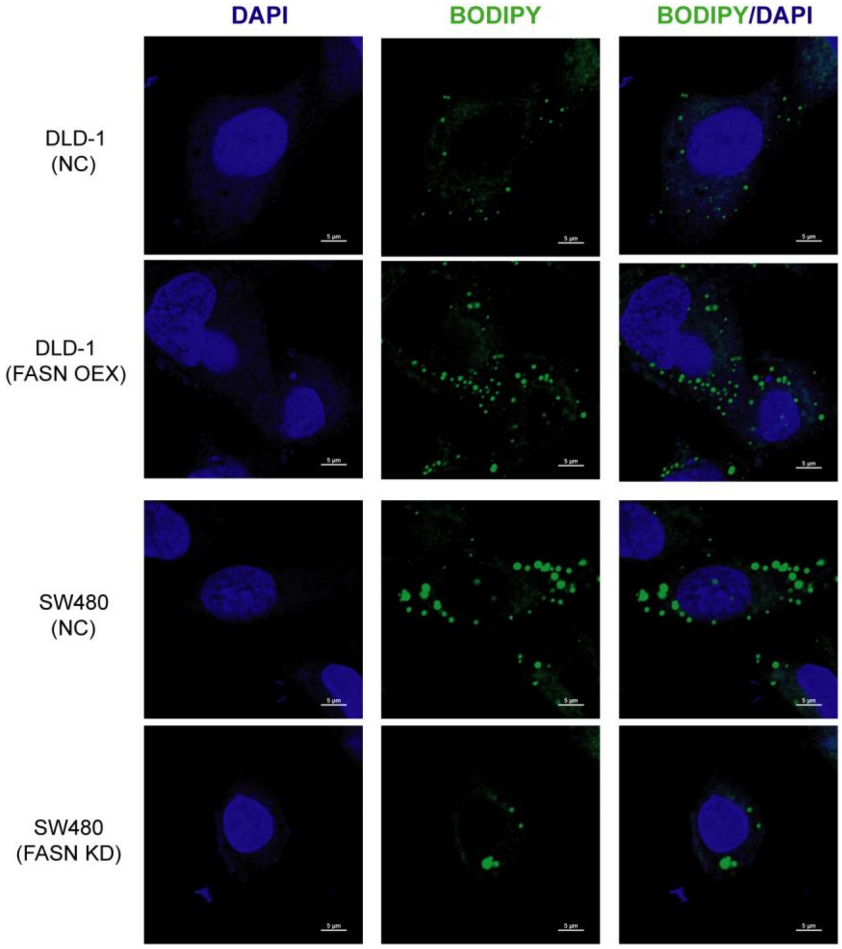

Figure. S16 Body weights of mice after antagomir-*EBV-miR-BART18-3p* injection.

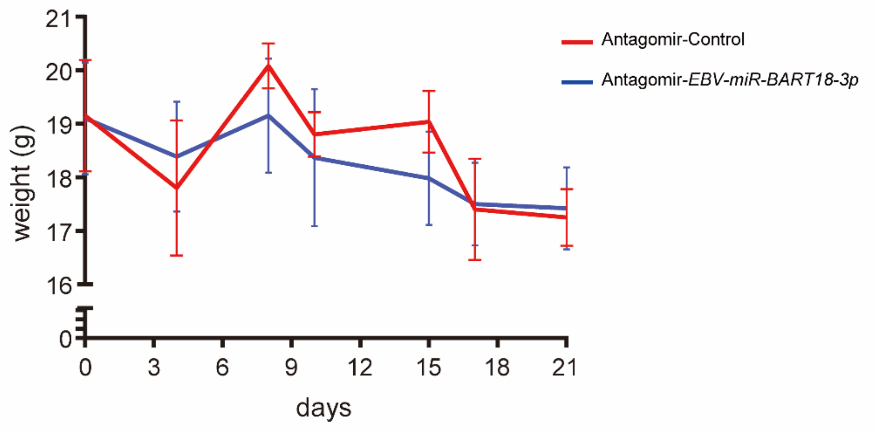

Supplementary tables

Table. S1 Demographic characteristics of CRC patients subjected to miRNA microarray analysis[2].

ID	Gender	Age	Diagnosis	Stage
1	Male	68	CRC	I
2	Male	73	CRC	I
3	Female	68	CRC	I
4	Male	62	CRC	II
5	Female	60	CRC	0
6	Female	59	CRC	II
7	Female	64	ADE	--
8	Male	60	ADE	--
9	Male	54	ADE	--
10	Male	59	ADE	--
11	Male	65	ADE	--
12	Female	52	ADE	--

CRC: colorectal cancer; ADE: colorectal adenoma

Table. S2 Fold change and *P* values of differentially expression microRNAs

**microRNA**	**CRC vs. ANT**		**CRC vs. ADE**	
	**Fold change**	***P* value**	**Fold change**	***P* value**
EBV-miR-BART18-3p	1.4705	4.43E-04	2.8895	8.48E-08
hsa-miR-135a-5p	5.0650	1.39E-03	2.1942	1.31E-02
hsa-miR-204-5p	2.7127	1.01E-02	-4.4540	2.18E-05

CRC: colorectal cancer; ANT: adjacent noncancerous tissue; ADE: colorectal adenoma

Table. S3 Demographic information of CRC and ADE patients for *EBV-miR-BART18-3p* expression PCR analysis.

Variables	Testing set (n = 322)		Validation set (n = 81)		Combined set (n = 403)	
	CRC (n%)	healthy donors (n%)	CRC (n%)	healthy donors (n%)	CRC (n%)	healthy donors (n%)
Age						
≤61	185 (57.5)	183 (56.8)	40 (49.4)	43 (53.1)	225 (55.8)	226 (56.1)
>61	137 (42.5)	139 (43.2)	41 (50.6)	38 (46.9)	178 (44.2)	177 (43.9)
Gender						
Male	167 (51.9)	157 (48.8)	34 (42.0)	43 (53.1)	201 (49.9)	200 (49.6)
Female	155 (48.1)	165 (51.2)	47 (58.0)	38 (46.9)	202 (50.1)	203 (50.4)
Location						
Colon	134 (41.6)		36 (44.4)		170 (42.2)	
Rectum	188 (58.4)		45 (55.6)		233 (57.8)	
Grade						
Low	131 (40.7)		38 (47.0)		169 (41.9)	
High	191 (59.3)		43 (53.0)		234 (58.1)	
Depth of invasion						
T1	7 (2.2)		4 (4.9)		11 (2.7)	
T2	69 (21.4)		36 (44.4)		105 (26.1)	
T3	88 (27.3)		19 (23.5)		107 (26.6)	
T4	158 (49.1)		22 (27.2)		180 (44.7)	
Lymph metastasis						
N0	125 (38.8)		31 (38.3)		156 (38.7)	
N1	197 (61.2)		50 (61.7)		247 (61.3)	
Distant metastasis						
M0	243 (75.5)		62 (76.5)		305 (75.7)	

M1	79 (24.5)	19 (23.5)	98 (24.3)
TNM			
I	42 (13.0)	11 (13.6)	53 (13.2)
II	72 (22.4)	20 (24.7)	92 (22.8)
III	129 (40.1)	31 (38.3)	160 (39.7)
IV	79 (24.5)	19 (23.4)	98 (24.3)

CRC: colorectal cancer; ADE: colorectal adenoma

Table.S4 Demographic information of CRC patients subjected to TMA[4].

Variables	Testing set (n = 862)		Validation set (n = 216)		Combined set （N=1078）	
	n	%	n	%	n	%
Age						
≤55	427	49.5	103	47.7	530	49.2
＞55	435	50.5	113	52.3	548	50.8
Gender						
Male	526	61.0	133	61.6	659	61.1
Female	336	39.0	83	38.4	419	38.9
Location						
Colon	379	44.0	96	44.4	475	44.1
Rectum	483	56.0	120	55.6	603	55.9
Grade						
Low	284	32.9	72	33.3	356	33.0
Intermediate/High	578	67.1	144	66.7	722	67.0
Depth of invasion						
T1	29	3.4	6	2.8	35	3.3
T2	191	22.2	53	24.5	244	22.6
T3	163	18.9	41	19.0	204	18.9
T4	479	55.6	116	53.7	595	55.2
Lymph node metastasis						
N0	491	57.0	130	60.2	621	57.6
N1	371	43.0	86	39.8	457	42.4
Distant metastasis						
M0	764	88.6	194	89.8	958	88.9
M1	98	11.4	22	10.2	120	11.1
TNM						
I	153	17.7	43	19.9	196	18.2
II	320	37.1	81	37.5	401	37.2
III	292	33.9	69	31.9	361	33.5
IV	97	11.3	23	10.7	120	11.1

Table. S5 Correlation between the expression of *miR-EBV-BART18-3p* in TMAs and selected clinicopathologic features

Variables [a]		Testing set (n = 839)			Validation set (n = 210)			Combined set (n = 1,049)		
		Low (%)	High (%)	P	Low (%)	High (%)	P	Low (%)	High (%)	P
All patients		491 (58.5)	348 (41.5)		128 (61.0)	82 (39.0)		619 (59.0)	430 (41.0)	
Age				0.0852			0.7874			0.0948
	≤55	230 (46.8)	184 (52.9)		60 (46.9)	40 (48.8)		290 (46.9)	224 (52.1)	
	>55	261 (53.2)	164 (47.1)		68 (53.1)	42 (51.2)		329 (53.2)	206 (47.9)	
Gender				0.4842			0.6360			0.405
	Males	293 (59.7)	216 (62.1)		77 (60.2)	52 (63.4)		370 (59.8)	268 (62.3)	
	Females	198 (40.3)	132 (37.9)		51 (39.8)	30 (36.6)		249 (40.2)	162 (37.7)	
Location				0.1566			0.3805			0.0977
	Colon	203 (41.3)	161 (46.3)		53 (41.4)	39 (47.6)		256 (41.4)	200 (46.5)	
	Rectal	288 (58.7)	187 (53.7)		75 (58.6)	43 (52.4)		363 (58.6)	230 (53.5)	
Grade				0.0095			0.2218			0.0042
	Low	143 (29.1)	131 (37.6)		38 (29.7)	31 (37.8)		181 (29.2)	162 (37.7)	
	Intermediate /High	348 (70.9)	217 (62.4)		90 (70.3)	51 (62.2)		438 (70.8)	268 (62.3)	
T				0.0006			0.2334			<.0001
	1	21 (4.3)	8 (2.3)		5 (3.9)	1 (1.2)		26 (4.2)	9 (2.1)	
	2	131 (26.7)	61 (17.5)		36 (28.1)	16 (19.5)		167 (27.0)	77 (17.9)	
	3	98 (20.0)	61 (17.5)		25 (19.5)	15 (18.3)		123 (19.9)	76 (17.7)	
	4	241 (49.1)	218 (62.6)		62 (48.4)	50 (61.0)		303 (49.0)	268 (62.3)	
N				<.0001			<.0001			<.0001
	0	399 (81.3)	89 (25.6)		106 (82.8)	22 (26.8)		505 (81.6)	111 (25.8)	
	1	92 (18.7)	259 (74.4)		22 (17.2)	60 (73.2)		114 (18.4)	319 (74.2)	

M				<.0001			<.0001			<.0001
	0	479 (97.6)	276 (79.3)		126 (98.4)	64 (78.0)		605 (97.7)	340 (79.1)	
	1	12 (2.4)	72 (20.7)		2 (1.6)	18 (22.0)		14 (2.3)	90 (20.9)	
TNM				<.0001			<.0001			<.0001
	I	131 (26.7)	21 (6.0)		38 (29.7)	6 (7.3)		169 (27.3)	27 (6.3)	
	II	264 (53.8)	53 (15.2)		68 (53.1)	12 (14.6)		332 (53.6)	65 (15.1)	
	III	83 (16.9)	202 (58.0)		21 (16.4)	46 (56.1)		104 (16.8)	248 (57.7)	
	IV	13 (2.6)	72 (20.7)		1 (0.8)	18 (22.0)		14 (2.3)	90 (20.9)	

[a] *Some cases lack information on the selected variables.*

Table. S6 Multivariate Cox regression analysis of *miR-EBV-BART18-3p* expression in TMAs and selected clinicopathologic variables predicting overall survival.

Variables	Testing set (n = 839)		Validation set (n = 210)		Combined set (n = 1,049)	
	Adjusted HR (95% CI) [a]	*P* [a]	Adjusted HR (95% CI) [a]	*P* [a]	Adjusted HR (95% CI) [a]	*P* [a]
Age (>55 VS. ≤55)	1.63 (1.36–1.96)	<.0001	2.23 (1.48–3.35)	0.0001	1.71 (1.45–2.02)	<.0001
Gender (Females VS. Males)	0.89 (0.74–1.06)	0.1963	0.79 (0.53–1.17)	0.2358	0.87 (0.74–1.03)	0.1
Location (Rectum VS. Colon)	0.82 (0.69–0.98)	0.0312	0.73 (0.50–1.07)	0.1045	0.80 (0.69–0.94)	0.0072
Grade (Intermediate/High VS. low)	0.86 (0.72–1.03)	0.1061	0.83 (0.56–1.25)	0.3764	0.86 (0.73–1.01)	0.0669
TNM (III/IV VS. I/II)	3.05 (2.46–3.78)	<.0001	4.24 (2.56–7.03)	<.0001	3.20 (2.63–3.89)	<.0001
EBV–miR-BART18-3p (High VS. Low)	3.72 (3.01–4.60)	<.0001	3.59 (2.21–5.83)	<.0001	3.72 (3.07–4.51)	<.0001

[a] Adjusted for age, gender, location (Colon or Rectum), grade (low VS. Intermediate/High) clinical stage (I/II VS. III/IV), and BART18-3p expression.

Table. S7 KEGG analysis of black module genes.

**Pathway name**	**Pathway source**	**Overlapping genes**	***P* value**
hypoxia-inducible factor pathway	BioCarta	CREB1; **LDHA**	0.000163
mRNA Processing	Wikipathways	SNRPD3; CLK4; RBM5	0.000518
HIF-1-alpha transcription factor network	PID	CREB1; **LDHA**	0.00284
TSH signaling pathway	Wikipathways	CREB1; TTF2	0.00321
Thyroid hormone synthesis - Homo sapiens (human)	KEGG	CREB1; TTF2	0.00381
inhibition of huntingtons disease neurodegeneration by histone deacetylase inhibitors	BioCarta	CREB1	0.00389
dermatan sulfate biosynthesis (late stages)	HumanCyc	CHST15	0.00519
lactate fermentation (reoxidation of cytosolic NADH)	HumanCyc	**LDHA**	0.00648
asparagine degradation	HumanCyc	AGA	0.00648
pyrimidine deoxyribonucleosides salvage	HumanCyc	TK2	0.00648

Table. S8 Expression of LDHA in TMAs and selected clinicopathologic features.

Variables		Testing set (n = 821)			Validation set (n = 209)			Combined set (n = 1,030)		
		Low (%)	High (%)	P	Low (%)	High (%)	P	Low (%)	High (%)	P
All patients		600 (73.1)	221 (26.9)		156 (74.7)	53 (25.3)		756 (73.4)	274 (26.6)	
Age				0.8393			0.6654			0.7132
	≤55	298 (49.7)	108 (48.9)		76 (48.7)	24 (45.3)		374 (49.5)	132 (48.2)	
	>55	302 (50.3)	113 (51.1)		80 (51.3)	29 (54.7)		382 (50.5)	142 (51.8)	
Gender				0.3152			0.8599			0.4085
	Males	368 (61.3)	127 (57.5)		95 (60.9)	33 (62.3)		463 (61.2)	160 (58.4)	
	Females	232 (38.7)	94 (42.5)		61 (39.1)	20 (37.7)		293 (38.8)	114 (41.6)	
Location				0.7860			0.9768			0.7944
	Colon	267 (44.5)	96 (43.4)		71 (45.5)	24 (45.3)		338 (44.7)	120 (43.8)	
	Rectal	333 (55.5)	125 (56.6)		85 (54.5)	29 (54.7)		418 (55.3)	154 (56.2)	
Grade				0.0144			0.5512			0.014
	Low	182 (30.3)	87 (39.4)		49 (31.4)	19 (35.8)		231 (30.6)	106 (38.7)	
	Intermediate/High	418 (69.7)	134 (60.6)		107 (68.6)	34 (64.2)		525 (69.4)	168 (61.3)	
T				0.0013			0.2155			0.0002
	1	21 (3.5)	5 (2.3)		5 (3.2)	1 (1.9)		26 (3.4)	6 (2.2)	
	2	147 (24.5)	34 (15.4)		41 (26.3)	9 (17.0)		188 (24.9)	43 (15.7)	
	3	119 (19.8)	33 (14.9)		33 (21.2)	8 (15.1)		152 (20.1)	41 (15.1)	
	4	313 (52.2)	149 (67.4)		77 (49.4)	35 (66.0)		390 (51.6)	184 (67.0)	
N				<.0001			<.0001			<.0001
	0	392 (65.3)	76 (34.4)		108 (69.2)	18 (34.0)		500 (66.1)	94 (34.3)	
	1	208 (34.7)	145 (65.6)		48 (30.8)	35 (66.0)		256 (33.9)	180 (65.7)	
M				0.0039			0.0520			0.0006
	0	545 (90.8)	185 (83.7)		144 (92.3)	44 (83.0)		689 (91.1)	229 (83.6)	
	1	55 (9.2)	36 (16.3)		12 (7.7)	9 (17.0)		67 (8.9)	45 (16.4)	
TNM				<.0001			0.0002			<.0001
	I	123 (20.5)	22 (10.0)		36 (23.1)	6 (11.3)		159 (21.0)	28 (10.2)	
	II	256 (42.7)	47 (21.3)		69 (44.2)	11 (20.8)		325 (43.0)	58 (21.2)	
	III	166 (27.7)	115 (52.0)		39 (25.0)	28 (52.7)		205 (27.1)	143 (52.2)	
	IV	55 (9.2)	37 (16.7)		12 (7.7)	8 (15.1)		67 (8.9)	45 (16.4)	

[a] *Some cases lack information on the selected variables.*

Table. S9 Multivariate Cox regression analysis of LDHA expression in TMAs and selected clinicopathologic variables predicting overall survival.

Variables	Testing set (n = 821)		Validation set (n = 209)		Combined set (n = 1,030)	
	Adjusted HR (95% CI) [a]	P [a]	Adjusted HR (95% CI) [a]	P [a]	Adjusted HR (95% CI) [a]	P [a]
Age (>55 VS. ≤55)	1.44 (1.21–1.72)	<.0001	1.82 (1.22–2.74)	0.0037	1.48 (1.26–1.74)	<.0001
Gender (Females VS. Males)	0.88 (0.73–1.06)	0.1831	0.68 (0.46–1.00)	0.0491	0.85 (0.72–1.00)	0.0455
Location (Rectum VS. Colon)	0.86 (0.72–1.03)	0.0913	0.60 (0.41–0.88)	0.0098	0.81 (0.69–0.95)	0.0107
Grade (Intermediate/High VS. Low)	0.88 (0.73–1.06)	0.1655	0.97 (0.65–1.45)	0.8987	0.89 (0.76–1.06)	0.1867
TNM (III/IV VS. I/II)	4.54 (3.73–5.53)	<.0001	7.04 (4.55–10.89)	<.0001	4.83 (4.04–5.78)	<.0001
LDHA (High VS. Low)	2.16 (1.79–2.61)	<.0001	1.59 (1.27–3.50)	<.0001	2.20 (1.85–2.61)	<.0001

[a] Adjusted for age, gender, location (Colon or Rectum), grade (low VS. Intermediate/High), clinical stage (I/II VS. III/IV) and LDHA expression.

Table. S10 KEGG enrichment of differentially expressed genes by RNA-seq assay

KEGG Term	ID	P-Value	Corrected P-Value	Input
AMPK signaling pathway	hsa04152	0.011508	0.161675	TSC2, **FASN**
Insulin signaling pathway	hsa04910	0.014637	0.161675	TSC2, **FASN**
Fatty acid biosynthesis	hsa00061	0.017935	0.161675	**FASN**
Collecting duct acid secretion	hsa04966	0.035558	0.161675	TCIRG1
Homologous recombination	hsa03440	0.036805	0.161675	XRCC2
Circadian rhythm	hsa04710	0.040537	0.161675	BHLHE40
ABC transporters	hsa02010	0.056547	0.161675	ABCA3
Fatty acid metabolism	hsa01212	0.060205	0.161675	**FASN**
Vibrio cholerae infection	hsa05110	0.068686	0.161675	TCIRG1
Acute myeloid leukemia	hsa05221	0.072298	0.161675	JUP
mTOR signaling pathway	hsa04150	0.075897	0.161675	TSC2
Synaptic vesicle cycle	hsa04721	0.079482	0.161675	TCIRG1
Epithelial cell signaling in Helicobacter pylori infection	hsa05120	0.085427	0.161675	TCIRG1
p53 signaling pathway	hsa04115	0.085427	0.161675	TSC2
PPAR signaling pathway	hsa03320	0.086612	0.161675	ANGPTL4
Arrhythmogenic right ventricular cardiomyopathy (ARVC)	hsa05412	0.092512	0.161897	JUP
Rheumatoid arthritis	hsa05323	0.1123	0.184965	TCIRG1
Thyroid hormone signaling pathway	hsa04919	0.143984	0.214007	TSC2
Lysosome	hsa04142	0.147314	0.214007	TCIRG1
Oxidative phosphorylation	hsa00190	0.159414	0.214007	TCIRG1
Ribosome	hsa03010	0.160505	0.214007	RPS28P7
Phagosome	hsa04145	0.183117	0.233058	TCIRG1
Transcriptional misregulation in cancer	hsa05202	0.208237	0.242943	JUP
Tuberculosis	hsa05152	0.208237	0.242943	TCIRG1
HTLV-I infection	hsa05166	0.288549	0.323175	ZFP36
Pathways in cancer	hsa05200	0.347408	0.374132	JUP
PI3K-Akt signaling pathway	hsa04151	0.364294	0.377786	TSC2
Metabolic pathways	hsa01100	0.469994	0.469994	TCIRG1, FASN

Table. S11 Expression of FASN in TMAs and selected clinicopathologic features

Variables		Testing set (n = 843)			Validation set (n = 199)			Combined set (n = 1,042)		
		Low (%)	High (%)	P	Low (%)	High (%)	P	Low (%)	High (%)	P
All patients		646 (77.5)	197 (22.5)		151 (75.9)	48 (24.1)		797 (76.5)	245 (23.5)	
Age				0.4994			0.7616			0.4571
	≤55	326 (50.5)	94 (47.7)		73 (48.3)	22 (45.8)		399 (50.1)	116 (47.4)	
	>55	320 (49.5)	103 (52.3)		78 (51.7)	26 (54.2)		398 (49.9)	129 (52.6)	
Gender				0.3758			0.6497			0.5266
	Males	491 (76.0)	123 (62.4)		92 (60.9)	31 (64.6)		483 (60.6)	154 (62.9)	
	Females	255 (39.5)	74 (37.6)		59 (39.1)	17 (35.4)		314 (39.4)	91 (37.1)	
Location				0.3066			0.2818			0.1641
	Colon	289 (44.7)	80 (40.6)		70 (46.4)	18 (37.5)		359 (45.0)	98 (40.0)	
	Rectal	357 (55.3)	117 (59.4)		81 (53.6)	30 (62.5)		438 (55.0)	147 (60.0)	
Grade				0.4063			0.7038			0.3612
	Low	209 (32.4)	70 (35.5)		49 (32.5)	17 (35.4)		258 (32.4)	87 (35.5)	
	Intermediate/High	437 (67.6)	127 (64.5)		102 (67.5)	31 (64.6)		539 (67.6)	158 (64.5)	
T				0.0022			0.4789			0.0007
	1	25 (3.9)	3 (1.5)		5 (3.3)	1 (2.1)		30 (3.8)	4 (1.6)	
	2	153 (23.7)	34 (17.3)		39 (25.8)	9 (18.8)		192 (24.1)	43 (17.6)	
	3	129 (20.0)	27 (13.7)		29 (19.2)	7 (14.6)		158 (19.8)	34 (13.9)	
	4	339 (52.5)	133 (67.5)		78 (51.7)	31 (64.6)		417 (52.3)	164 (66.9)	
N				<.0001			<.0001			<.0001
	0	430 (66.6)	47 (23.9)		105 (69.5)	13 (27.1)		535 (67.1)	60 (24.5)	
	1	216 (33.4)	150 (76.1)		46 (30.5)	35 (72.9)		262 (32.9)	185 (75.5)	
M				<.0001			0.0026			<.0001
	0	596 (92.3)	142 (72.1)		140 (92.7)	37 (77.1)		736 (92.4)	189 (77.1)	

	1	50 (7.7)	45 (22.8)		11 (7.3)	11 (22.9)		61 (7.6)	56 (22.9)	
TNM				<.0001			<.0001			<.0001
	I	138 (21.4)	10 (5.1)		36 (23.8)	4 (8.3)		174 (21.8)	14 (5.7)	
	II	282 (43.7)	28 (14.2)		67 (44.4)	7 (14.6)		349 (43.8)	35 (14.3)	
	III	176 (27.2)	113 (57.4)		37 (24.5)	27 (56.3)		213 (26.7)	140 (57.1)	
	IV	50 (7.7)	46 (23.4)		11 (7.3)	10 (20.8)		61 (7.7)	56 (22.9)	

[a] *Some cases lack information on the selected variables.*

Table. S12 Multivariate cox regression analysis of FASN expression in TMAs and selected clinicopathologic variables predicting overall survival

Variables	Testing set (n = 843)		Validation set (n = 199)		Combined set (n = 1,042)	
	Adjusted HR (95%	P [a]	Adjusted HR (95%	P [a]	Adjusted HR (95%	P [a]
Age (>55 VS. ≤55)	1.37 (1.15–1.64)	0.0005	1.95 (1.31–2.91)	0.001	1.44 (1.23–1.70)	<.000
Gender (Females VS.	0.93 (0.77–1.11)	0.4106	0.74 (0.50–1.08)	0.1145	0.89 (0.76–1.05)	0.1671
Location (Rectum VS.	0.83 (0.69–0.99)	0.0378	0.61 (0.42–0.89)	0.0104	0.79 (0.67–0.93)	0.0039
Grade (Intermediate/High	0.88 (0.73–1.06)	0.1676	0.84 (0.57–1.24)	0.3813	0.88 (0.74–1.03)	0.1169
TNM (III/IV VS. I/II)	4.77 (3.92–5.81)	<.000	6.98 (4.56–10.68)	<.000	5.04 (4.22–6.01)	<.000
FASN (High VS. Low)	2.20 (1.83–2.64)	<.000	2.07 (1.40–3.06)	0.0003	2.19 (1.85–2.58)	<.000

[a] Adjusted for age, gender, location (colon or rectum), grade (low VS. intermediate/high), clinical stage (I/II or III/IV) and FASN expression.

Table. S13 Demographic characteristics of CRC patients and healthy donors subjected to EBV ELISA assays.

Variables	CRC (n%)	Healthy donors (n%)
Age		
≤61	142 (55.7)	145 (57.1)
>61	113 (44.3)	109 (42.9)
Gender		
Male	151 (59.2)	150 (59.1)
Female	104 (40.8)	104 (40.9)
Location		
Colon	97 (38.0)	
Rectum	158 (62.0)	
Grade		
Low	85 (33.3)	
High	170 (66.7)	
Depth of invasion		
T1	6 (2.4)	
T2	65 (25.5)	
T3	79 (31.0)	
T4	105 (41.2)	
Lymph metastasis		
N0	93 (36.5)	
N1	162 (63.5)	
Distant metastasis		
M0	196 (76.9)	
M1	59 (23.1)	
TNM		
I	23 (9.0)	
II	67 (26.3)	
III	106 (41.6)	
IV	59 (23.1)	

Table. S14 Clinical characteristics of the patients used for developing PDX models

Models	Sex	Age (years)	7th Edition AJCC	Location	Diagnosis
CRC-11	Female	48	III	Rectal	CRC
CRC-12	Male	52	III	Rectal	CRC
CRC-14	Female	50	III	Rectal	CRC
CRC-16	Male	61	III	Colon	CRC
CRC-19	Female	71	III	Colon	CRC
CRC-21	Female	66	III	Colon	CRC
CRC-23	Male	51	III	Colon	CRC
